# Does a third intermediate model for the vomeronasal processing of information exist? Insights from the macropodid neuroanatomy

**DOI:** 10.1007/s00429-021-02425-2

**Published:** 2021-11-20

**Authors:** Mateo V. Torres, Irene Ortiz-Leal, Paula R. Villamayor, Andrea Ferreiro, José Luis Rois, Pablo Sanchez-Quinteiro

**Affiliations:** 1 Department of Anatomy, Animal Production and Clinical Veterinary Sciences, Faculty of Veterinary, University of Santiago de Compostela, Av Carballo Calero s/n, 27002 Lugo, Spain; 2 Department of Zoology Genetics and Physical Anthropology, Faculty of Veterinary, University of Santiago de Compostela, Lugo, Spain; 3 Marcelle Nature Park, Outeiro de Rei, Lugo, Spain

**Keywords:** Wallaby, Marsupials, Vomeronasal, Accessory olfactory bulb, G proteins, Immunohistochemistry

## Abstract

The study of the α-subunit of Gi2 and Go proteins in the accessory olfactory bulb (AOB) was crucial for the identification of the two main families of vomeronasal receptors, V1R and V2R. Both families are expressed in the rodent and lagomorph AOBs, according to a segregated model characterized by topographical anteroposterior zonation. Many mammal species have suffered from the deterioration of the Gαo pathway and are categorized as belonging to the uniform model. This scenario has been complicated by characterization of the AOB in the tammar wallaby, *Notamacropus eugenii*, which appears to follow a third model of vomeronasal organization featuring exclusive Gαo protein expression, referred to as the intermediate model, which has not yet been replicated in any other species. Our morphofunctional study of the vomeronasal system (VNS) in Bennett’s wallaby, *Notamacropus rufogriseus,* provides further information regarding this third model of vomeronasal transduction. A comprehensive histological, lectin, and immunohistochemical study of the Bennett’s wallaby VNS was performed. Anti-Gαo and anti-Gαi2 antibodies were particularly useful because they labeled the transduction cascade of V2R and V1R receptors, respectively. Both G proteins showed canonical immunohistochemical labeling in the vomeronasal organ and the AOB, consistent with the anterior–posterior zonation of the segregated model. The lectin *Ulex europaeus* agglutinin selectively labeled the anterior AOB, providing additional evidence for the segregation of vomeronasal information in the wallaby. Overall, the VNS of the Bennett’s wallaby shows a degree of differentiation and histochemical and neurochemical diversity comparable to species with greater VNS development. The existence of the third intermediate type in vomeronasal information processing reported in *Notamacropus eugenii* is not supported by our lectin-histochemical and immunohistochemical findings in *Notamacropus rufogriseus*.

**Supplementary Information:**

The online version contains supplementary material available at 10.1007/s00429-021-02425-2.

## Introduction

The sense organs transmit physical (sight, hearing, and touch) or chemical (smell and taste) signals. In particular, the processing regions associated with smell are anatomically connected to the limbic system, and smell is, therefore, intimately linked to memories, emotions, and behavior (Müller-Schwarze 2006). Parallel to the olfactory system is the vomeronasal or accessory olfactory system, and the vomeronasal organ (VNO), which is the primary organ involved in this system (Wysocki and Lepri 1991; Halpern and Martínez Marcos 2003), is also found in the nose, specialized in mediating innate behaviors, as territorial aggression (Chamero et al. 2007) and maternal behavior (Keverne 2002).

Morphologically, the vomeronasal system (VNS) is primarily comprised of the VNO, the vomeronasal nerves (NVNs), and the accessory olfactory bulbs (AOB). In the 1970s, the first evidence was presented to indicate that this system was specialized for the detection of pheromones (Powers and Winans 1975), which are substances that establish chemical communication between individuals of the same species; pheromone-based communication does not reflect the simple transmission of information, as occurs in the main olfactory system, but rather serves as an interaction that elicits some type of non-conscious physiological or behavioral change in the receiver (Wyatt 2014). Pheromones modulate important aspects of reproduction (Martín-Sánchez et al. 2015; Demir et al. 2020), the adaptive response to stress (Papes et al. 2010; Pallé et al. 2019), and the social behaviors of animals (Ibarra-Soria et al. 2014; Cross et al. 2021). Thus, to intervene in chemical communications, a solid morphofunctional foundation of the VNS is necessary to allow for the understanding of the mechanism of action mediated by various pheromones in different species. These attempts have been hampered by the broad morphofunctional (Salazar et al. 2007; Stoyanov et al. 2021), molecular (Isogai et al. 2011; Katreddi and Forni 2021), and genomic (Grus et al. 2005; Villamayor et al. 2021) diversity of this system among mammals, which makes the establishment of a valid general pattern challenging, even among species belonging to the same order, such as Rodentia (Torres et al. 2020).

The diversity of G protein alpha (Gα) subunit expression has been the subject of specific studies that were crucial to identifying the vomeronasal receptor (VR) family. The expression of Gαi and Gαo proteins were both first detected in the AOB by Shinohara et al. (1992), who observed that nerve endings of vomeronasal neuroreceptor cells innervating the AOB were organized into two regions: one rich in Gαi2 and the other rich in Gαo. This heterogeneous pattern was restricted to the nerve and glomerular layers of the AOB and was conserved in the NVNs. Determining the involvement of both G proteins in the signal transduction chain of VNO neuroreceptor cells was key to the identification of the two main VR families. First, 3 years after the identification of Gα protein expression in the AOB, a family of receptors, named V1R, was discovered in mice (Dulac and Axel 1995), and the pattern of V1R expression in the VNO coincided with the pattern of Gαi2 expression. Parallel studies examining Gαi2 and Gαo expression in both the VNO and the AOB, using immunohistochemistry in opossum (Halpern et al. 1995) and mouse (Jia and Halpern 1996) specimens and in situ hybridization in mice (Berghard and Buck 1996), demonstrated that both proteins are expressed in separate subpopulations of VNO neurons, which suggested the existence of two independent sensory transduction pathways. However, the second G protein receptor was not identified until 1997, when three different studies examining the expression of Gαo in the VNO simultaneously demonstrated the existence of a second VR family, named V2R (Herrada and Dulac 1997; Matsunami and Buck 1997; Ryba and Tindirelli 1997). Since then, the immunohistochemical characterization of Gαi2 and Gαo has been considered to serve as excellent phenotypic indicators of V1R and V2R expression in the VNS, respectively.

Initially, all mammals were thought to segregate vomeronasal sensory information into two neuronal subsets, determined by the expression patterns of the V1R and V2R receptors. However, the immunohistochemical study of the goat VNS did not identify Gαo-immunoreactivity in either the VNO or in the NVN layer of the AOB (Takigami et al. 2000), indicating the existence of a different projection pattern for vomeronasal neurons to the AOB in some mammals and highlighting the importance of performing comparative studies of the VNS in multiple species. Thus, studies performed across a range of mammals as allowed for the conclusion that two types of VNS organization exist. The segregated type, in which the VN neurons express both G proteins, Gαi2 and Gαo, is observed in Rodents, such as mouse, rat, octodon (Suarez and Mpodozis 2009), guinea pig (Takigami et al. 2004), and capybara (Suarez et al. 2011b; Torres et al. 2020); Lagomorpha, such as rabbit (Villamayor et al. 2018); Marsupials, including opossum (Halpern et al. 1995); and tenrecs (Suarez et al. 2009). The uniform type, in which the VN neurons only express a single G protein subtype, specifically the Gαi2 subunit, can be observed in goat (Takigami et al. 2000), sheep (Salazar et al. 2007), dog (Salazar et al. 2013), horse, marmoset, musk shrew (Takigami et al. 2004), squirrel, hyrax (Suarez et al. 2011a), and hippopotamus (Kondoh et al. 2017b).

The absence of the V2R pathway in the uniform model has been hypothesized to be closely related to the appearance of sexual dimorphism in mammals (Suarez et al. 2011a). This deterioration has become a challenge for understanding the role played by the VNS in mammals, and this complex panorama was further convoluted by the appearance of a study of the VNS in the marsupial tammar wallaby, *Notamacropus eugenii* (Schneider et al. 2012). This species presents a VNS organization similar to the uniform type, with the crucial difference that only immunopositivity against Gαo was identified, becoming the first species displaying a new, third type of vomeronasal pheromone transduction pathway organization, in which only the V2R pathway is conserved.

Since the publication of this study, no contributions to the literature have appeared that can either support or refute the existence of this third model, which remains an open question that must be viewed cautiously. The authors themselves presented some reasonable doubt; although they did not identify Gαi2 immunopositivity in the VNO and AOB, they did identify functional V1R receptor-encoding genes within the tammar wallaby genome (Young et al. 2010). Although this suggested the existence of V1R receptor-expressing cells in the tammar wallaby VNS, V1R receptors could also be expressed in other tissues, such as the V1R expression observed in the rabbit testis (Xia et al. 2020; Villamayor et al. 2021).

To examine the potential existence of this third model of VNS organization, we performed a morphological and immunohistochemical study of the VNS of Bennett’s wallaby, focusing on the expression of Gα subunits in the VNO and AOB. Additionally, we performed a comprehensive histological, lectin-histochemical, and immunohistochemical investigation of the entire VNS, employing antibodies against microtubule-associated protein 2 (MAP-2), glial fibrillary acidic protein (GFAP), growth-associated protein 43 (GAP43), calbindin, calretinin, olfactory marker protein (OMP), and the lectins *Ulex europaeus* agglutinin (UEA) and *Lycopersicon esculentum* agglutinin (LEA).

## Materials and methods

Three adult individuals of Bennett's Wallaby (*Notamacropus rufogriseus*) used in our study were transferred by Marcelle Natural Park (Outeiro de Rei, Lugo) after dying of natural causes. After separating the heads from the carcass, the skin, jaws, tongue and eyes were dissected out, and a window opened in the dorsal part of the cranium. The fixatives used were 10% Formol and Bouin's fluid. In the latter, after 24 h, the samples were transferred to 70% alcohol.

### Samples extraction

The following anatomical structures: nasal cavity (NC), vomeronasal organs (VNO), nasal septum mucosa and the olfactory bulbs (OB), were employed in the histological study. Additionally, a successful cannulation of the incisor canal was performed in one of the specimens.

#### Nasal cavity and vomeronasal organs

The entire NC was separated through a transverse incision made rostral to the ethmoidal fossa with the help of a rotary saw. In one individual, the resulting sample was destined to study the macroscopic and microscopic changes in the VNO topography. In a second one, after opening the NC laterally, the nasal conchae were removed. This allowed visualization of the entire nasal septum, where the VNO were identified on both sides of the base of the nasal septum. They were extracted under a surgical microscope, Zeiss OPMI 1 Ent.

#### Main and accessory olfactory bulbs

The olfactory bulbs are located deep in the ethmoidal fossa. To access them, with the help of a gouge forceps the bone was carefully removed from the orbital fossa, which laterally covers the bulbs. Then, the dura mater and olfactory nerves were dissected out, separating them with a scalpel from the cribriform ethmoid plate.

### Samples processing for histological study

All samples were embedded in paraffin, except one NC that previously was decalcified in TBD-1 Shandon Decalcifier (Thermo, Pittsburgh, PA) continuously stirred for 9 days. Then, a series of equidistant transversal cuts were made in the NC from the tip of the nose to the caudal end of the vomeronasal cartilage, obtaining five blocks. They were photographed and after that, all the blocks were embedded in paraffin.

#### Cutting

With the help of a Leica Reichert Jung microtome, the paraffin blocks were cut with a thickness ranging from 5 to 8 mm, employing the thinner sections in the study of the VNO and the thicker one to the study of the AOB.

### General histological stainings

We employed the following general histological stains: hematoxylin–eosin (HE), periodic acid-Schiff (PAS) and Alcian Blue (AB) for neutral and acidic mucopolysaccharides, respectively, and Nissl staining for nervous tissue. In addition, two other specific stains were performed:

#### Gallego’s trichrome

Useful for the differentiation of the components of the connective tissue. It stains both erythrocytes and muscle fibers green, collagen light blue, epithelium and glandular tissue red, bone dark blue and cartilage purple. The protocol used by us has been detailed in Ortiz-Leal et al. (2020).

#### Tolivia

This technique allows to discriminate black myelinated nerve fibers and pink neuronal somas. The protocol followed by us is detailed in Villamayor et al. (2020).

### Histochemical and immunohistochemical staining

A detailed account of the procedures is given in Torres et al. (2020).

#### Histochemical labeling with lectins (HQ)

Lectins are naturally occurring carbohydrate-binding molecule, that can be used to detect glycoconjugates in tissues. We have used two lectins; LEA, that comes from tomato, *Lycopersicon esculentum*, and recognizes *N*-acetyl-glucosamine (Salazar and Sanchez-Quinteiro 2003), and UEA that comes from gorse, *Ulex europaeus*, and labels the l-fucose pathway (Alroy et al. 1986).

The protocol for the LEA is as follows. (i) The tissular endogenous peroxidase activity is blocked to avoid interference with the final developing step by incubating in a 3% H_2_O_2_ solution for 10 min. (ii) Sections are incubated for 30 min at room temperature in 2% bovine serum albumin (BSA), which prevents nonspecific binding. (iii) The sections were incubated overnight (4 °C) in biotinylated LEA diluted in 0.5% BSA. The following day, samples were incubated (iv) for 90 min at room temperature in Vectastain ABC complex (Vector Laboratories, Burlingame, CA, USA). Finally, (v) the sections were developed by incubating the sections in a solution of 0.05% diaminobenzidine (DAB) and 0.003% H_2_O_2_ for 5–10 min until the desired color reaction is observed when monitored with the microscope.

The protocol for UEA began with the same first two steps. Subsequently, (iii) incubation with UEA lectin is carried out for 1 h at room temperature followed by (iv) 3 washes of 5 min in 0.1 M phosphate buffer (PB, pH 7.2), and (v) sections afterwards incubated for 12 h (4 °C) in a peroxidase conjugated immunoglobulin against UEA. Finally, the samples were developed (vi) by incubation in the same DAB solution as the UEA.

#### Immunohistochemical techniques (IHQ)

The first step was (i) to block endogenous peroxidase activity. Then, (ii) non-specific binding was blocked with 2.5% normal horse serum from the ImmPRESS Anti-Mouse IgG/Anti-rabbit IgG Reagent Kit (Vector Laboratories, CA, USA) for 30 min. (iii) The primary antibody was then added to the corresponding dilution (Table 1) and allowed to incubate overnight (4 °C). The next day, (iv) the samples were incubated for 20 min at room temperature with the corresponding ImmPRESS VR Polymer HRP anti-rabbit IgG reagent. Finally, and after (v) rinsing in Tris buffer (pH 7.61) for 10 min, (vi) the samples were finally developed using DAB as a chromogen in the same way as for lectins.

**Table 1:** Lectins and antibodies used. Species of elaboration, manufacturer, dilutions, catalog number

Ab/lectin	1st Ab species/dilution	1st Ab catalogue number	2nd Ab species/dilution/catalogue number
Anti-Gαo	Rabbit 1:100	MBL 551	ImmPRESS VR HRP Anti-Rabbit IgG Reagent MP-6401-15
Anti-Gαi2	Rabbit 1:100	Sta Cruz Biotechnology SC-7276	ImmPRESS VR HRP Anti-Rabbit IgG Reagent MP-6401-15
Anti-OMP	Goat 1:400	Wako S44-10001	Horse 1:250 Vector BA-9500
Anti-MAP2	Mouse 1:200	Sigma M4403	ImmPRESS VR HRP Anti-Mouse IgG Reagent MP-6402-15
Anti-GAP43	Mouse 1:800	Sigma G9264	ImmPRESS VR HRP Anti-Mouse IgG Reagent MP-6402-15
Anti-GFAP	Rabbit 1:400	Dako Z0334	ImmPRESS VR HRP Anti-Rabbit IgG Reagent MP-6401-15
Anti-CB	Rabbit 1:6000	Swant CB38	ImmPRESS VR HRP Anti-rabbit IgG Reagent MP-6401-15
Anti-CR	Rabbit 1:6000	Swant 7697	ImmPRESS VR HRP Anti-rabbit IgG Reagent MP-6401-15
UEA-I	1:10	Vector L-1060	Rabbit 1:50 DAKO P289
LEA	20 μg/ml	Vector B-1175	Vectastain ABC reagent PK-4000

All immunohistochemical protocols were run with the appropriate controls. In the absence of a positive control from wallabies, we reproduced the whole histochemical procedure with mouse tissues known to express the respective antigens. Samples for which the primary antibody was replaced by antibody diluent were used as negative controls. For lectins, controls were performed both without the addition of lectins and with the preabsorption of lectins, using an excess amount of the corresponding sugar.

### Confocal laser scanning microscopy

The autofluorescence imaging of elastic fibres of the VNO was performed by confocal laser scanning microscopy. After cryopreserving the samples in 30% sucrose, they were cut in a sliding microtome at 40 μm thickness, and stored in 0.1 M PB, pH 7.2, at 4 °C until free-floating processing. Sections were counterstained with TO-PRO-3 iodide (1 μM; Molecular Probes) for 15 min and mounted in Slow-Fade solution (Molecular Probes Cat. # S7461). A Bio-Rad MRC 1024 ES setup for dual-channel fluorescence using green autofluorescence and TO-PRO-3 iodide (far red fluorescence) filter settings (100 mW Ar laser, wavelength 488 nm, and He–Ne laser, wavelength 633 nm, respectively) were used. Images were taken using a 20 × lens (NA 0.45) on a Nikon TE 2000 inverted microscope, with separate emission filtering for each type of fluorescence (RIAIDT, University of Santiago de Compostela, Spain).

### Acquisition of images and digital treatment

Images were taken using the Karl Zeiss Axiocam MRc5 digital camera coupled to a Zeiss Axiophot microscope. If needed, Adobe Photoshop CS4 (Adobe Systems, San Jose, CA) was used to adjust parameters such as contrast or brightness, balance light levels, and crop or resize images for presentation in this work. No specific characteristics within the images were altered, enhanced, moved or introduced.

## Results

### Macroscopic study

**Fig. 1:**
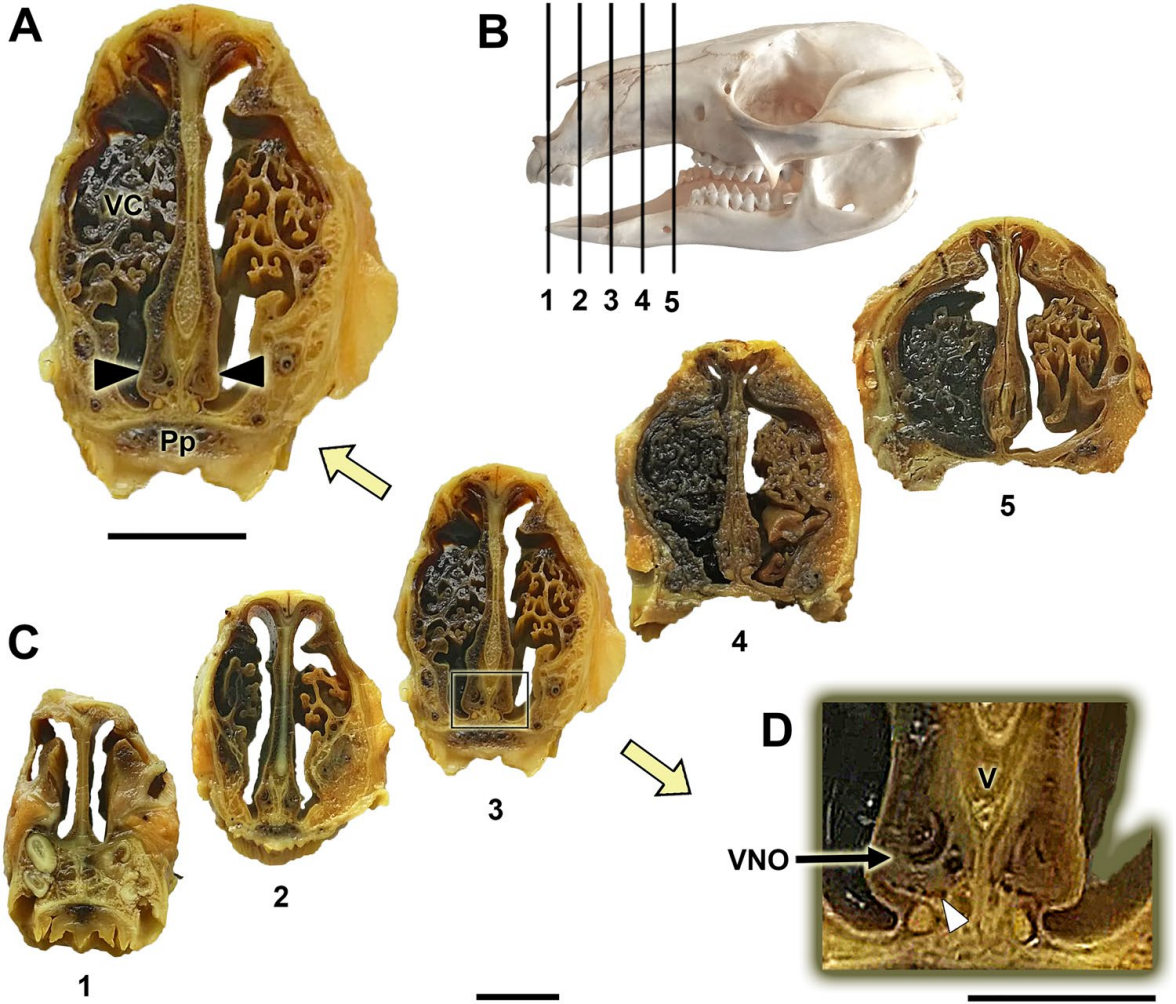
Cross-sections of the Bennett’s wallaby nasal cavity in five levels (C) ordered from rostral (1) to caudal (5). The levels of the sections are represented in the lateral view of the skull **(B).** The central part of the vomeronasal organ (VNO) is located at levels 3 and 4, and level 3 can be observed at higher magnification in **(A)**. The VNO occupies the ventral recess of the nasal cavity on both sides of the nasal septum (black arrowheads). **(D)** Higher magnification of the inset in (C, level 3) showing the VNO. The dorsal crest of the palatine process forms a lateral projection that supports the ventral part of the VNO (white arrowhead). Pp: Palatine process; V: Vomer; VC: Ventral concha. Scale bars: (A, C) 1 cm; (D) 0.5 cm

**Fig. 2:**
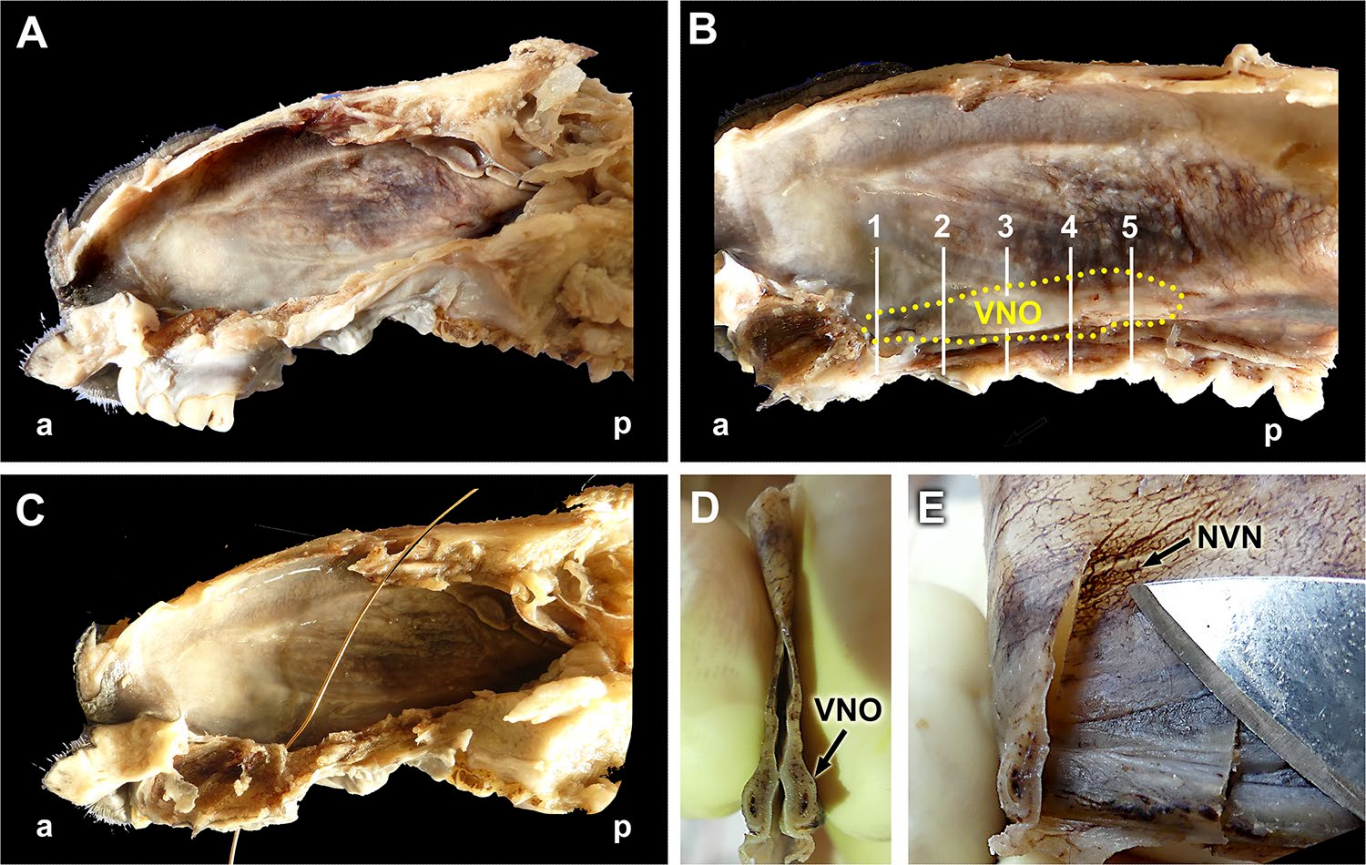
Dissection of the Bennett’s wallaby VNO. **(A)** Dissection plane after the removal of the lateral wall of the nasal cavity and the left nasal conchae. **(B)** A more advanced plane of dissection, from which the roof, floor, and incisor and canine teeth roots have been removed to delimit the projection area of the vomeronasal organ (VNO, dashed yellow line). The levels of the five sections described in Fig. 1 are indicated. **(C)** Cannulation through the nasopalatine duct demonstrates the functionality of the oro-nasal communication. **(D)** Cross section of the organ after extraction, showing its slender shape and the central vomeronasal duct. **(E)** By observing the translucent respiratory mucosa of the nasal cavity, the vomeronasal nerves (NVN, arrow) can be recognized. a: anterior; p: posterior; NVN: Vomeronasal nerves; VNO: Vomeronasal organ

**Fig. 3:**
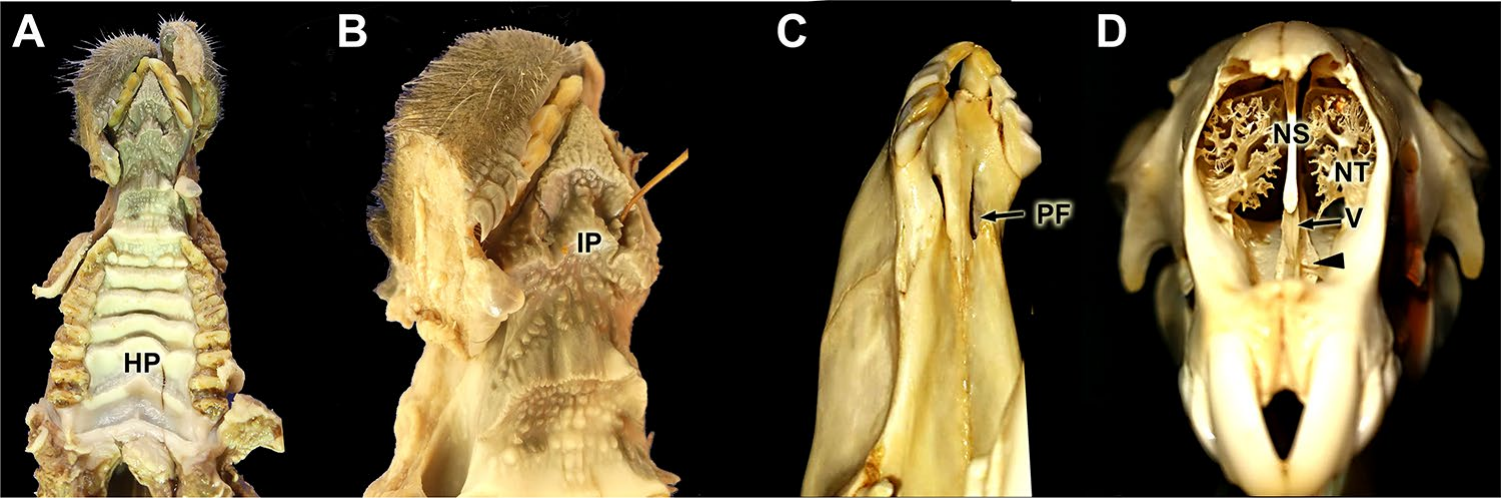
Demonstration of the communication between the oral and nasal cavities. **(A)** Ventral view of the hard palate (HP). **(B)** Ventral view of the passage through the incisive papilla (IP) of the cannula showed in Fig. 2C. **(C)** Ventrolateral view of the bony palate showing the palatal fissure (PF). **(D)** Anterior view of the nasal cavity, in which the vomer bone (V), the cartilage of the nasal septum (NS), and the nasal turbinates (NT) are visible. The location of the vomeronasal organ (VNO) is indicated (arrowhead)

The primary anatomical landmarks identified for Bennett’s wallaby VNS are shown in Figs. 1, 2, 3. Cross-sections made along the entire length of the decalcified nasal cavity allowed us to define the exact location of the VNO within the anterior half of the nasal cavity and to identify topographic relationships (Fig. 1A–D). The VNO occupies the ventral recess of the cavity, lying on both sides of the nasal septum. Its shape is elongated in the dorsoventral axis and relatively narrow in the lateromedial direction, especially in the more caudal part of the organ. This shape is proportional to the dorsoventrally elongated nasal septum of this species (Fig. 1A). The medial side of the VNO lays over the lateral lamina of the vomer bone, and the ventral portion of the VNO rests on the continuous ridge formed by the palatine processes of the incisive and maxillary bones. The latter forms two thin lateral projections in its dorsal crest that widen the surface of contact with the organ (Fig. 1D).

The dissection and extraction of both complete VNOs were aided by the information obtained from the cross-sectional study. Two longitudinal sections of the nasal cavity, one dorsal section, along the left nasal bone, and one ventral section, in a left parasagittal plane, allowed for the removal of the lateral nasal cavity wall and the nasal turbinates that originate from the nasal cavity wall, making the nasal septum visible (Fig. 2A, B). The nasopalatine duct could then be progressively cannulated from the nasal cavity (Figs. 2C, Fig. 3A, B) to the oral cavity due to the presence of a functional palatal fissure, as demonstrated in the skull (Fig. 3C). The thickness and pigmentation of the nasal mucosa prevented the identification of the projection area of the VNO or the NVN path (Fig. 3B). Progressively lifting the mucosa in the rostro-caudal direction, separating the mucosa from the nasal septum, allowed access to the VNO, which occupies the curved lateral recess formed by the vomer bone (Fig. 3D). Once the VNOs were dissected out, their nature was confirmed by cross-sectional examination (Fig. 2D). Additionally, the observation of the mucosa against the light allowed us to visualize the branches of the NVNs (Fig. 2E).

**Fig. 4:**
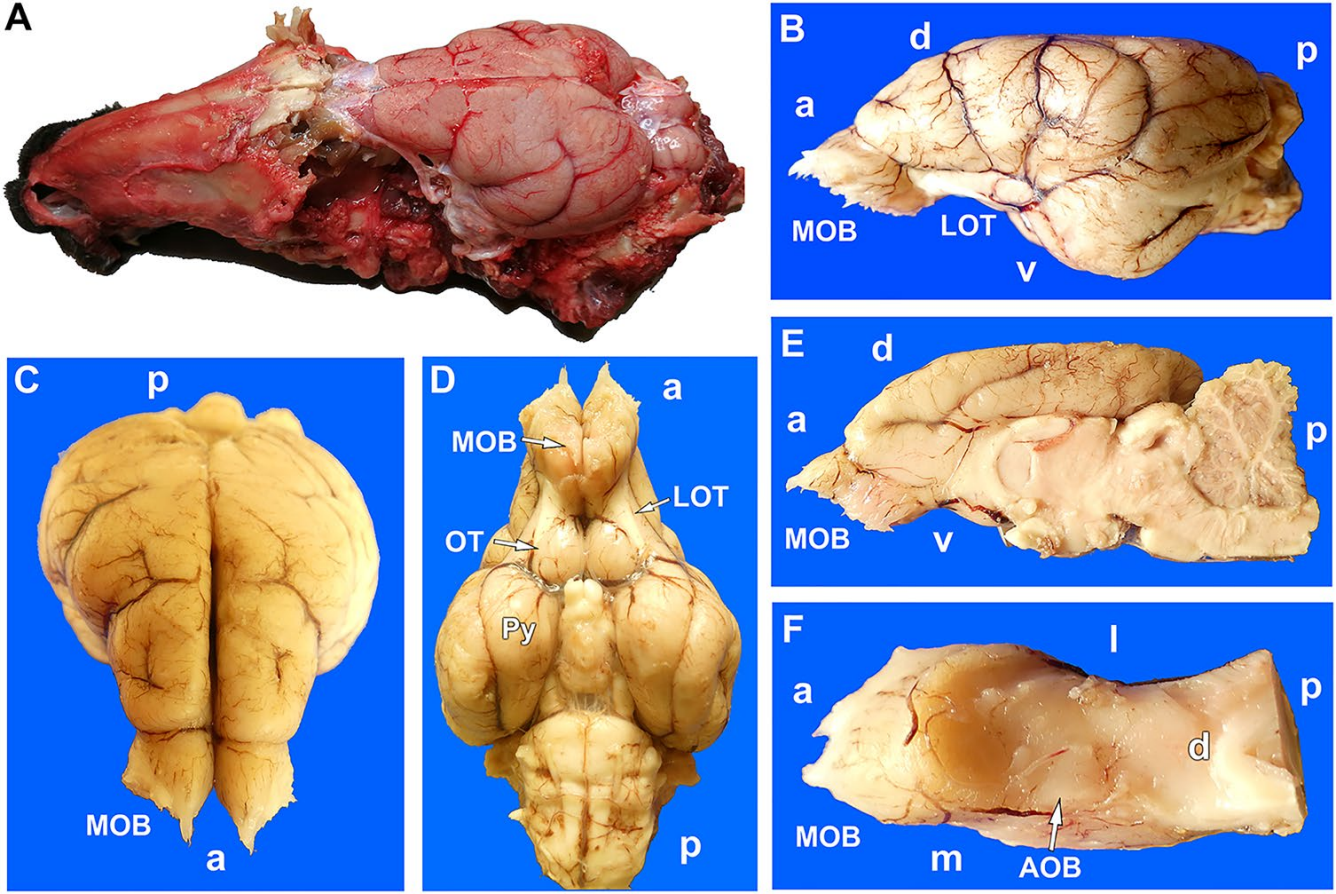
Bennett’s wallaby olfactory brain. **(A)** Dorsolateral view of the skull after the removal of the vault and side walls. The olfactory bulbs, in situ, are covered by the dura mater and are mostly hidden in the ethmoidal fossa. **(B)** Lateral, **(C)** anterodorsal, and **(D)** ventral views of the encephalon. In the ventral view, the remarkable development of the olfactory tubercles (OT) and the piriform lobes (Py) can be observed. **(E)** Medial view of the right hemiencephalon. **(F)** Once the cerebral hemisphere has been removed, in the dorsal view of the right olfactory bulb, an elevation, surrounded by blood vessels that corresponds to the accessory olfactory bulb (AOB) is observed medially. MOB, main olfactory bulb; LOT, lateral olfactory tubercles; directionality is indicated by the following: a, anterior; p, posterior; d, dorsal; v, ventral

To extract the whole brain, the lateral walls of the cranium were removed using a rotary saw and gouge forceps, completely revealing both hemiencephalons (Fig. 4A). The olfactory bulbs are housed in the narrow and slender ethmoidal fossa. The bulbs, as well as the rest of the rhinencephalon, olfactory tracts and tubercula, and piriform lobes, are well developed in this species (Fig. 4B–E). The AOB is easily recognizable in the dorsocaudal part of the olfactory bulb (Fig. 4F), because it is delimited by vascular formations. The arrival of the NVN is appreciable by its medial border.

### Microscopic study

**Fig. 5:**
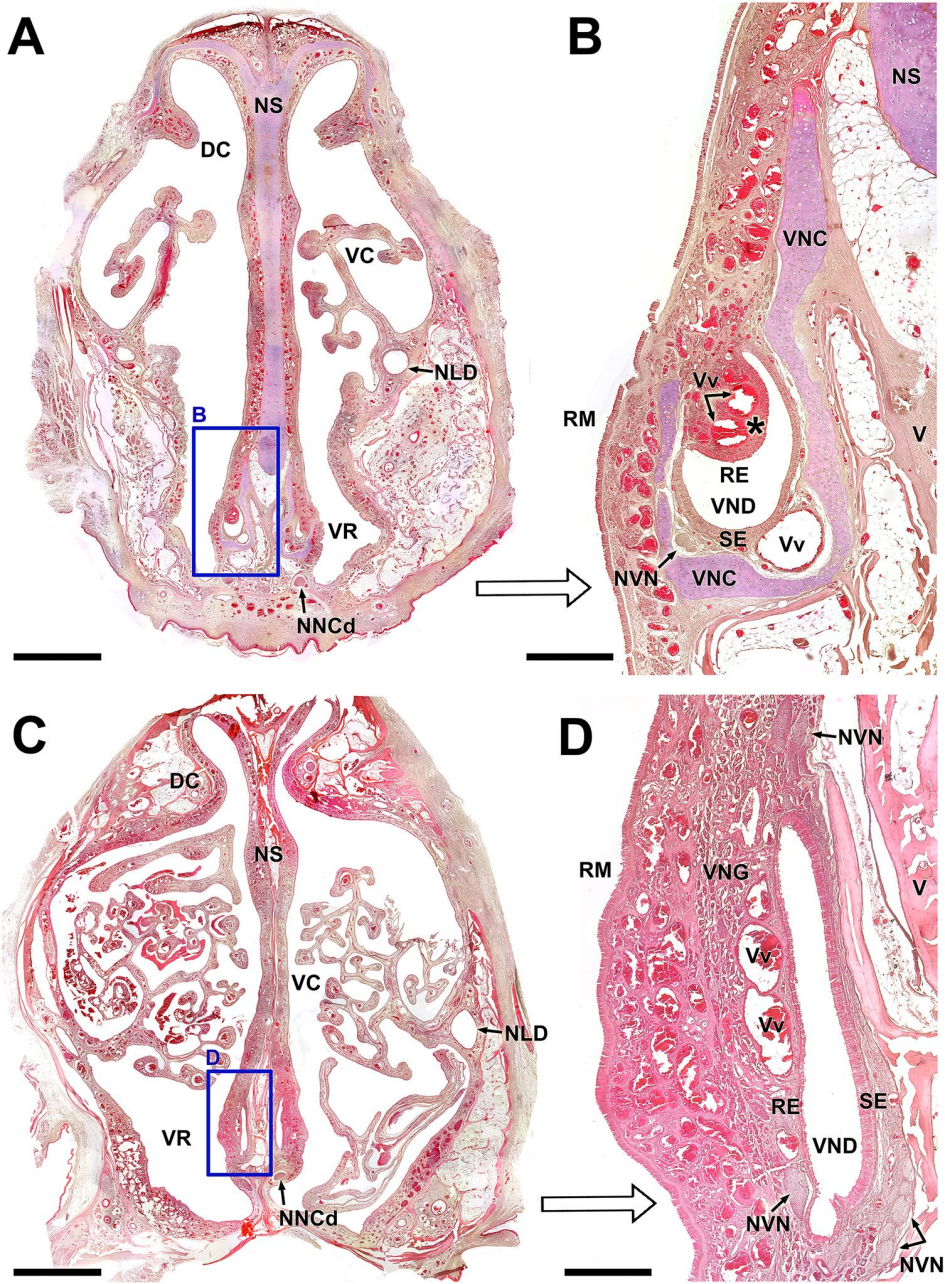
Decalcified microscopic transverse sections of the Bennett’s wallaby VNO. **A** Cross section of the anterior region of the nasal cavity (Level 2 in Fig. 1), showing the vomeronasal organs (VNOs) located in the ventral recess. The right VNO is framed and shown at a higher magnification in (**B**). At this level, the J-shaped vomeronasal cartilage (VNC) and the crescent-shaped vomeronasal duct (VND) are the primary landmarks of the organ. The lateral parenchyma forms a recognizable mushroom body (asterisk), containing two large veins (Vv) filled with blood in this sample. Together with the large ventromedial vein, they are responsible for the pumping mechanism of the organ. **C** Cross section of the central region of the nasal cavity (Level 4 in Fig. 1). The right VNO, shown in greater detail in (**D**), is located at the base of the nasal septum (NS), dorsal to the caudal nasal nerve passage. The VND is no longer crescent shaped, but dorsoventrally elongated, with a highly developed sensory neuroepithelium (SE) on the medial side. Remarkably, at this level, the VNO lacks any cartilaginous capsule. The amount of glandular tissue (VNG) is much greater, and new bundles of vomeronasal nerves (NVN) appear in the ventral and dorsal areas of the organ. DC: Dorsal concha; NLD: Nasolacrimal duct; NS: Nasal septum; RE: Respiratory epithelium; RM: Respiratory mucosa; V: Vomer bone; VC: Ventral concha. Stain: Hematoxylin–eosin. Scale bars: (A,C) 500 μm; (B,D) 150 μm

Histological, decalcified cross-sections of the whole snout from Bennett’s wallaby confirmed the location of the VNO in the ventral recess of the nasal cavity at the base of the nasal septum (Fig. 5A). The VNO of the Bennet’s wallaby presents all the necessary components to perform its function in a highly developed fashion: a cartilaginous capsule (vomeronasal cartilage, VNC) and a vomeronasal duct (VND) associated with a soft tissue rich in veins, nerves, and glands. The organization of all these components varies noticeably along the rostrocaudal axis. Anteriorly (Fig. 5A, B), the cartilage is J-shaped, and the duct has a crescent or semilunar-like shape. The sensory neuroepithelium is arranged along the medial concave surface of the lumen, whereas the respiratory epithelium occupies a lateral location that covers the lateral luminal surface of the duct. The lateral soft tissue forms a recognizable mushroom body containing two or three large veins (Vv).

At a more caudal level (Fig. 5C, D), several significant changes can be observed in the structure of the VNO. The VND becomes more slender and elongated dorsoventrally, and remarkably, the VNC disappears completely. Therefore, the boundaries between the parenchyma and the respiratory mucosa of the nasal cavity are more diffuse. At both levels, the nasal mucosa is highly vascularized, comprising a dense network of subepithelial and periglandular capillaries.

**Fig. 6:**
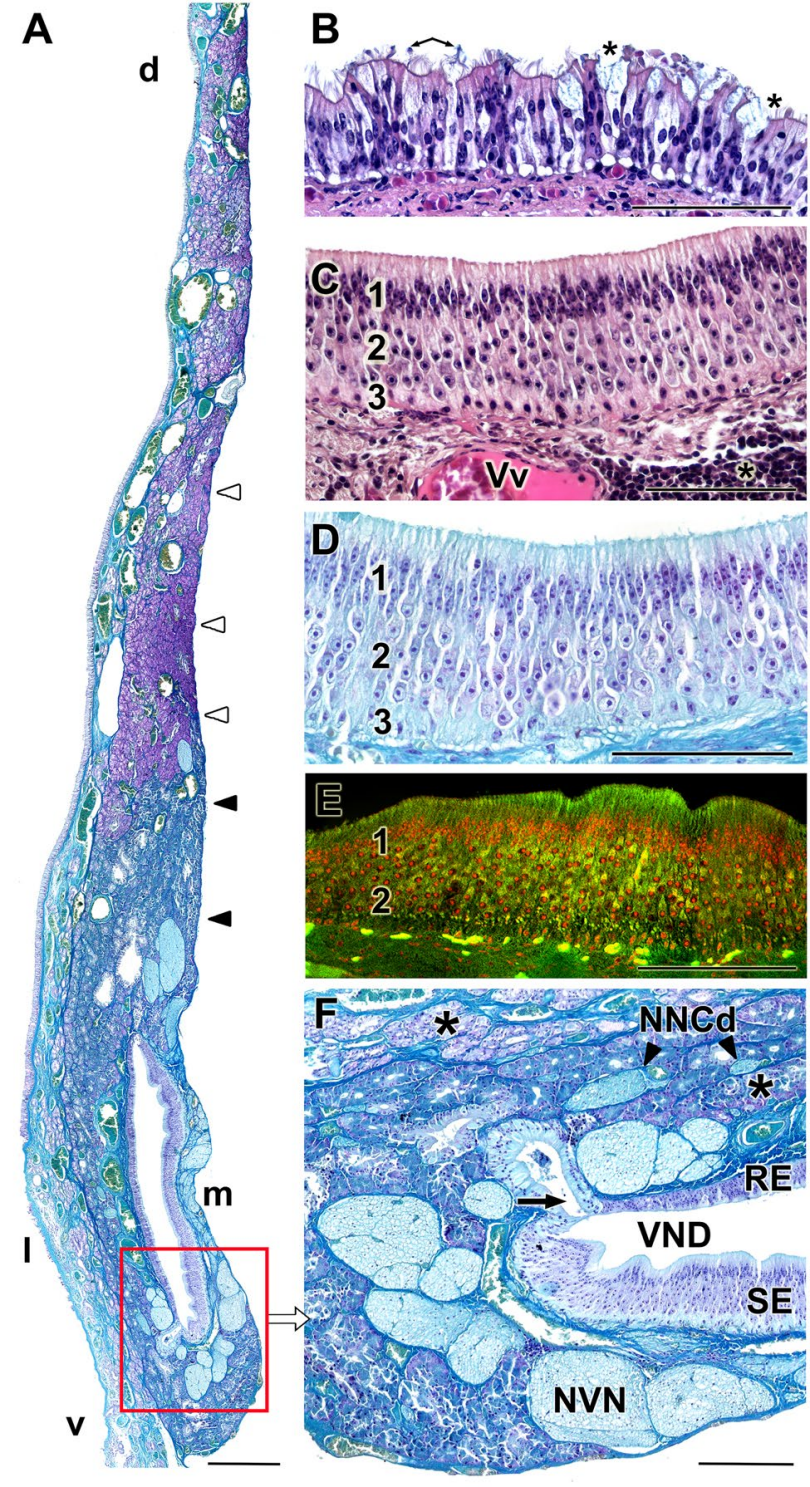
Histology of the vomeronasal organ in the Bennett’s wallaby. **A** Transverse section of the vomeronasal organ (VNO, level 4 in Fig. 1), showing the high level of development of the medial sensory epithelium (SE) and the number and thickness of the nerve trunks, arranged medially (m), dorsally (d) and ventrally (v) to the vomeronasal duct (VND). At this level, the glandular tissue is distributed along the entire medial part of the parenchyma, but with a much higher density dorsally to the VND. In this dorsal area, Gallego’s staining allows for the differentiation of two compartments. The uppermost compartment (white arrowheads) is strongly stained with fuchsin, whereas the lower compartment (black arrowheads), closer to the VND, is weakly stained. **B** Respiratory epithelium (RE) of the VNO, containing goblet cells (asterisks) and cilia (arrows). **C** VNO neuroreceptor epithelium. The three main strata are differentiated: sustentacular cells (1), neuroreceptor cells (2), and basal cells (3). In the lamina propria, a vein (Vv) of great caliber and diffuse lymphoid tissue (asterisk) is visible. **D** Gallego's trichrome staining makes it possible to differentiate the cell edges and the characteristics of the nuclei. **E** Autofluorescence confocal microscopy of the sensory epithelium allows for the clear differentiation of the zones corresponding to the main strata (same numbering as in C and D). **F** Ventral commissure of the VND, in which the large opening of a gland (arrow), profuse myelin innervation corresponding to the vomeronasal nerves (NVN), and several additional branches of the caudal nasal nerve (NNCd, arrowheads) in direct contact with the vomeronasal glands (asterisks), are noticeable. RE: Respiratory epithelium; SE: Sensory epithelium; VND: Vomeronasal duct; d: dorsal; l: lateral; m: medial; v: ventral. Stains: (A, D, F) Gallego’s Trichrome; (B, C) Hematoxylin–eosin; (E) Confocal microscopy. Scale bars: (A) 250 µm; (B-F) 100 µm

**Fig. 7:**
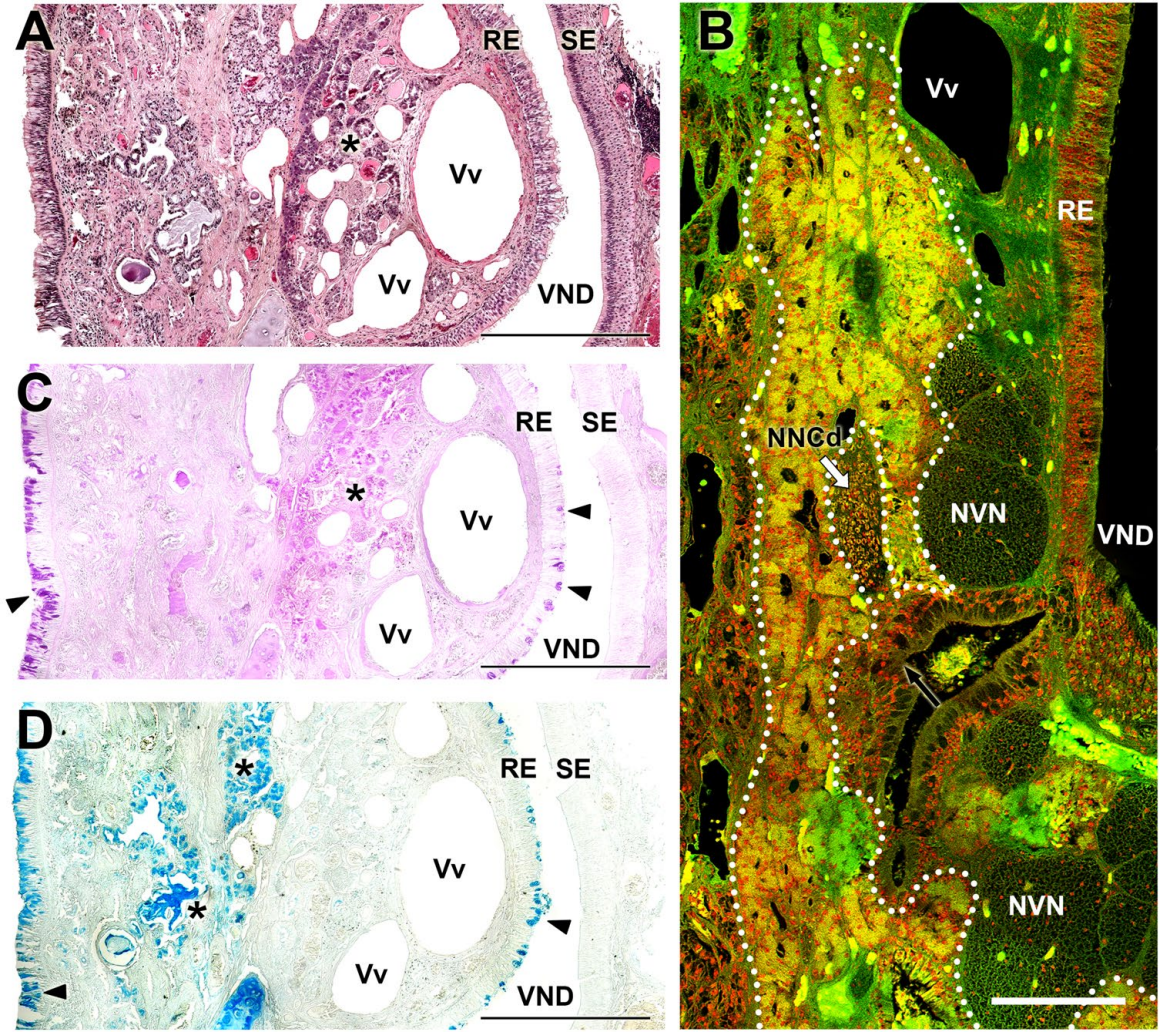
Glandular component of the wallaby VNO. **A, C, and D** Consecutive serial sections of the anterior portion of the vomeronasal organ (VNO, at level 2 in Fig. 1), stained with (A) hematoxylin–eosin, (C) periodic acid–Schiff (PAS), and (D) Alcian blue. The PAS-positive glands are concentrated in the lateral parenchyma surrounding the venous sinuses, characteristic of this region (asterisk in C). In the lamina propria of the respiratory mucosa, just lateral to the VNO there are tubulo-acinar Alcian blue–positive glands (asterisks in D). The goblet cells of the respiratory epithelium (RE) contain secretions that are positive for both stains (arrowheads in C and D). **B** Confocal microscopy of the left area framed in Fig. 8B makes it possible to characterize the development of the glandular components in a more caudal level of the VNO (white dashed line). Remarkably, a large myelinic branch of the nasal caudal nerve (NNcD, white arrow) is associated with the glands. NVN: Vomeronasal nerve; RE: Respiratory epithelium; SE: Sensory epithelium; VND: Vomeronasal duct. Stains: (A), Hematoxylin–eosin; (B), Confocal; (C), PAS; (D), Alcian blue. Scale bars: (A, C, D) 500 µm; (B) 100 µm

**Fig. 8:**
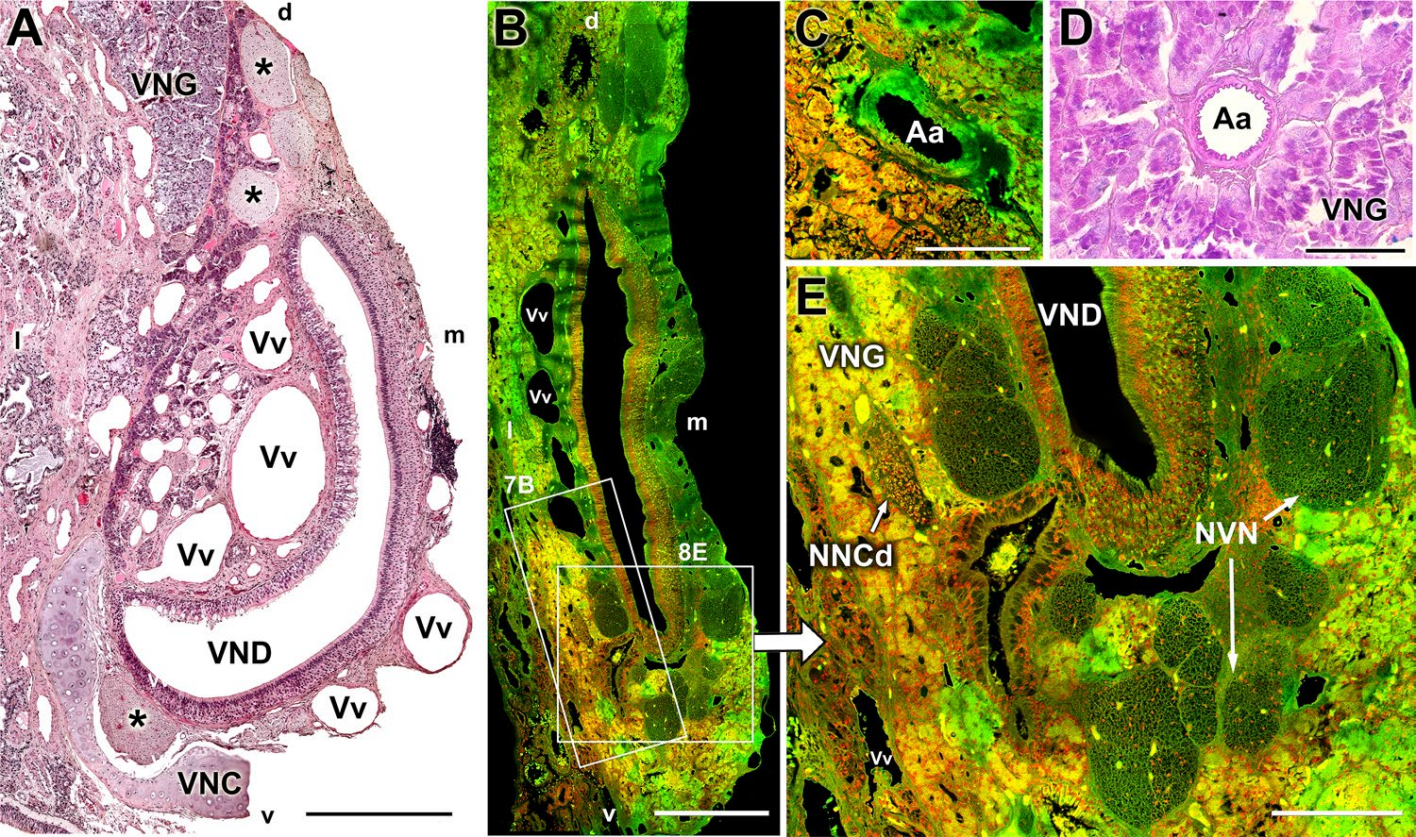
Vascularization of the wallaby VNO. (**A)** In the anterior segment of the vomeronasal organ (VNO, Level 3 in Fig. 1), the presence of venous (Vv) sinuses stands out, which are distributed both in the elevation of the lateral part of the parenchyma and in the lamina propria of the medial mucosa. Vomeronasal nerves (NVN, asterisks) are located in both the dorsal and ventral commissures of the vomeronasal duct (VND). **(B)** Confocal microscopic autofluorescence image at a more caudal level (Level 4 in Fig. 1), showing the development of the lateral venous sinuses. **(C)** In a more caudal level (Level 5 in Fig. 1), autofluorescence allows for the identification of arteries (Aa) by their elastic tunic. **(D)** At the same caudal level of the VNO, the arteries are surrounded by periodic acid-Schiff (PAS)-positive glandular tissue. **(E)** Enlargement of the right box in B, showing the densely packed branches of the vomeronasal nerves and a branch of the nasal caudal nerve (NNCd) in the midst of vomeronasal glands (VNG). VNC: Vomeronasal cartilage; VND: Vomeronasal duct; d, dorsal; l, lateral; m, medial; v, ventral. Stains: (A) Hematoxylin–eosin; (D) PAS; (B, C, E) Confocal microscopy. Scale bars: (A) 500 µm; (B) 250 μm; (C, D, E) 100 µm

The microscopic study of the dissected VNO was performed using histological stains and confocal microscopy on decalcified sections. The results are shown in Figs. 6, 7, 8. The lateral respiratory epithelium (Fig. 6A, B) has a ciliated pseudostratified appearance, with a small number of pseudolayers. An abundant number of periodic acid–Schiff (PAS)-positive and Alcian blue–positive goblet cells can be observed (Fig. 7C, D), similar to those that cover the respiratory mucosa of the nasal cavity. The columnar cells show a variable affinity for hematoxylin–eosin dyes, including strongly basophilic cells and others that are hardly stained. The medial neuroreceptor epithelium shows remarkable development, even though the receptor cells are not as densely packed as in other species. The receptor cells feature rounded nuclei that are variably stained by hematoxylin (Fig. 6C).

The cell limits are very well defined, to the point that the dendritic knobs approaching the lumen of the VND and the axonal projections toward the lamina propria are neatly differentiated. Gallego’s trichrome stain (Fig. 6A, D) enhances the visualization of the nuclear morphology, the high development of the connective tissue in the lamina propria, and the profuse network of sensorial NVNs. The NVNs not only rest under the sensorial epithelium but also extend dorsally and ventrally around the two commissures of the duct and beyond. Atypically, the NVNs partially occupy the lamina propria of the VNO respiratory epithelium (Fig. 6F). Autofluorescence confocal microscopy allows for the recognition of the layers that form the receptor epithelium, which appears as bands: basal, receptor, and sustentacular cells and the zone of the cell processes (Fig. 6E). Additionally, the lamina propria of the sensory epithelium in the Bennet’s wallaby has uniquely large veins and a huge accumulation of diffuse lymphoid tissue (Fig. 6C).

The glandular tissue within the VNO parenchyma is highly developed, surrounding the VND primarily on the lateral side. In the anterior zone of the VNO (level 2), the VNO parenchyma is concentrated in the elevated portion within the lateral mucosa, associated with the large veins that give it shape. The nature of this glandular tissue is tubulo-acinar (Fig. 7A), comprising mucoserous acini with PAS-positive and Alcian blue–negative secretions (Fig. 7C, D, respectively). Although this area is also rich in serous acinar Alcian blue–positive PAS-negative glands (Fig. 7D), they are associated with the respiratory mucosa of the nasal cavity. The caliciform cells in the respiratory epithelium of the VNO are both PAS- and Alcian blue–positive (Fig. 7C, D).

Caudally, in the central zone of the VNO, the lateral vomeronasal glands (VNG) decreases, and mucoserous acinar tissues invade the ventral and dorsal areas of the parenchyma (Fig. 6A, F). The morphology of the ventral glands is more visible in the confocal study (Fig. 7B), which reveals acini with a neat central cavity. Dorsally, the glands form large patches of glandular tissue that can be differentially stained using Gallego’s trichrome stain (Fig. 6A); the lower patch, closer to the VND, is weakly stained by fuchsin, whereas the dorsal patch is strongly stained by fuchsin. Additionally, the ventral patches are primarily PAS-positive, whereas the dorsal patches are Alcian blue–positive (Suppl. Fig. 1). In the most caudal part of the VNO, coinciding with the blind sac of the VND, a huge density of PAS-positive serous glands can be observed (Fig. 8D, Suppl. Fig. 2).

The development of the NVN branches in the parenchyma is striking. From the anterior segment of the VNO, these unmyelinated branches join in large fascicles located dorsally, medially, and even ventrally to the duct (Figs. 6A, F, 7B, 8A, E). An important vegetative innervation from the autonomic nervous system was also observed, formed by myelinated fibers and derived from the caudal nasal nerve. These nerve bundles are located exclusively in the lateral parenchyma and are always closely related to the VNG and the veinous (Figs. 6A, F, 7B, 8A, E) structures that they innervate.

The vascular component of the parenchyma primarily consists of large veins and venous sinuses that give the VNO the appearance typical of erectile tissue, especially in the anterior part of the duct (Fig. 8A). The presence of two or three large-caliber veins in the lamina propria of the medial sensory epithelium is remarkable (Fig. 8A). Arterial vessels in both the anterior and central levels are difficult to identify, even when using confocal microscopy, which typically allows for the visualization of the elastic tunic of the arteries due to the autofluorescent nature of elastin (Fig. 8C). However, in the most caudal portion of the organ, the caliber of the veins decreases (Fig. 7B), and the arterial vessels responsible for providing the blood supply to the large anterior venous network are noticeable (Fig. 8C, D).

**Fig. 9:**
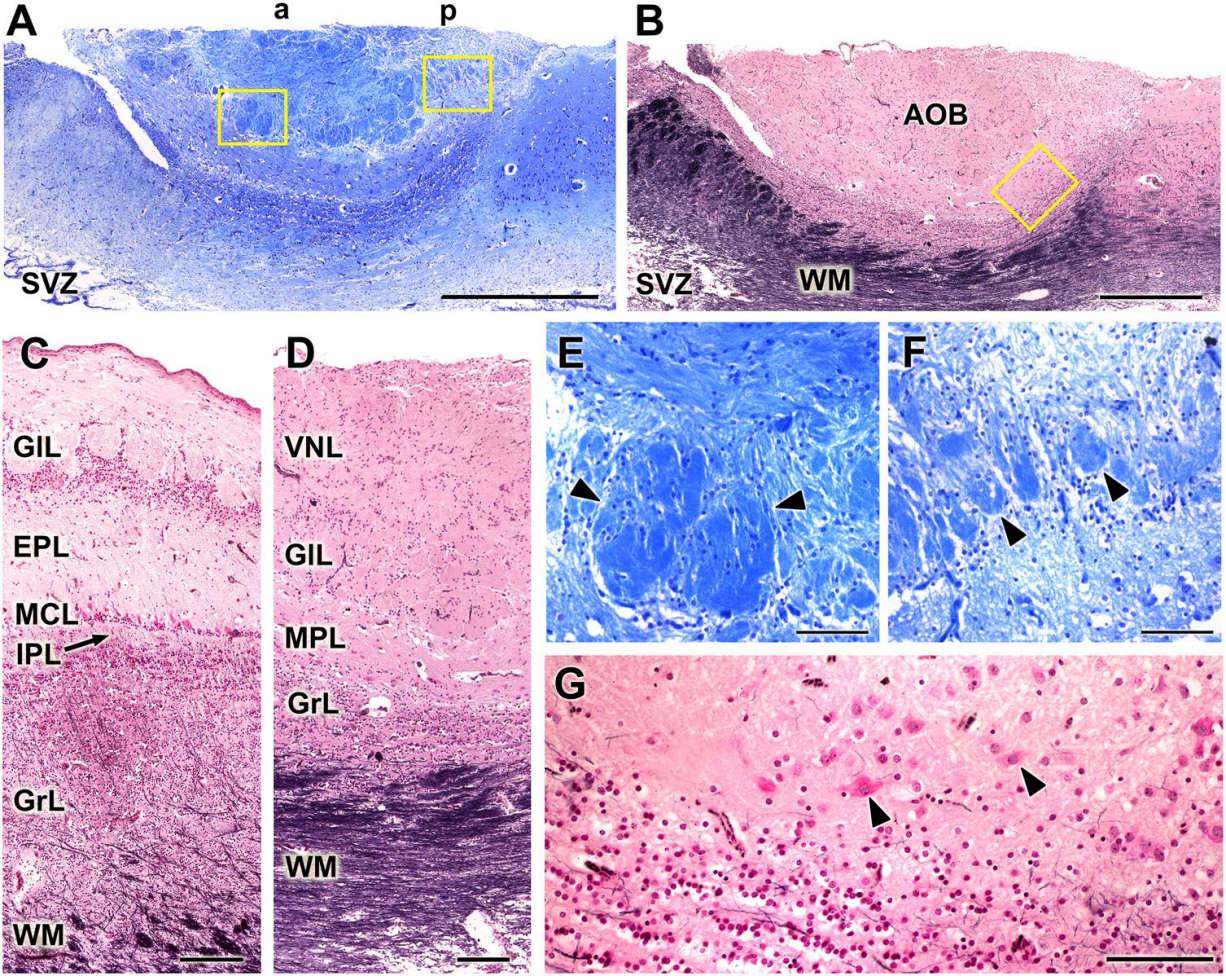
Histological study of the Bennett’s Wallaby AOB. **(A)** Low power sagittal section of the accessory olfactory bulb (AOB). Two zones are discriminated, an anterior (a) zone and a posterior (p) zone. **(B)** The Tolivia stain reveals the great development of the white matter (WM) pathways that leave the olfactory bulb (OB) from the main OB (MOB) and the AOB. **(C and D)** Comparison of the lamination between MOB (C) and the AOB (D). It is remarkable how, in the AOB, the plexiform (IPL) and mitral (MCL) layers are fused into a mitral-plexiform layer (MPL). **(E and F)** Higher magnification of the insets in A. A noticeable difference in size can be observed between the glomeruli (arrowheads) of both zones of the AOB: the anterior glomeruli (E) are much larger than the glomeruli on the posterior segment (F). **(G)** Higher magnification view of the inset in B, showing the deep layers of the AOB. Mitral cells (black arrow) are scattered in the MPL. EPL: External plexiform layer; GlL: Glomerular layer; GrL: Granular layer; IPL: Internal plexiform layer; MCL: Mitral cell layer; VNL: Vomeronasal nerve layer; SVZ: Subventricular zone. Stains: (A, E, F), Nissl; (B, C, D, G), Tolivia. Scale bars: (A, B) 1 mm; (C, D) 250 μm; (E, F, G) 100 µm

Microscopically, the AOB shows an appreciable development, which matches the noticeable macroscopic appearance (Fig. 9A, B). Structurally, the AOB shows a well-differentiated laminar organization (Fig. 9D). The nerve and glomerular layers of the AOB are highly developed, similar to the development observed in the MOB. However, in the MOB, the mitral cell and plexiform layers are distinctly separated (Fig. 9C), whereas in the AOB, they reorganize into a joint mitral-plexiform layer, which contains diffusely arranged mitral cells (Fig. 9D). The mitral cells have a fusiform or polyhedric shape and are easily recognized by Tolivia staining (Fig. 9G).

Another remarkable feature in the microscopic organization of the wallaby AOB is the appreciable difference in size between the larger glomeruli of the anterior part and the smaller glomeruli in the posterior segment (Fig. 9E, F) and the great development of the white matter bundles that delimit a granular zone of relatively scarce development (Fig. 9B, D).

### Immunohistochemical and lectin-histochemical study

**Fig. 10:**
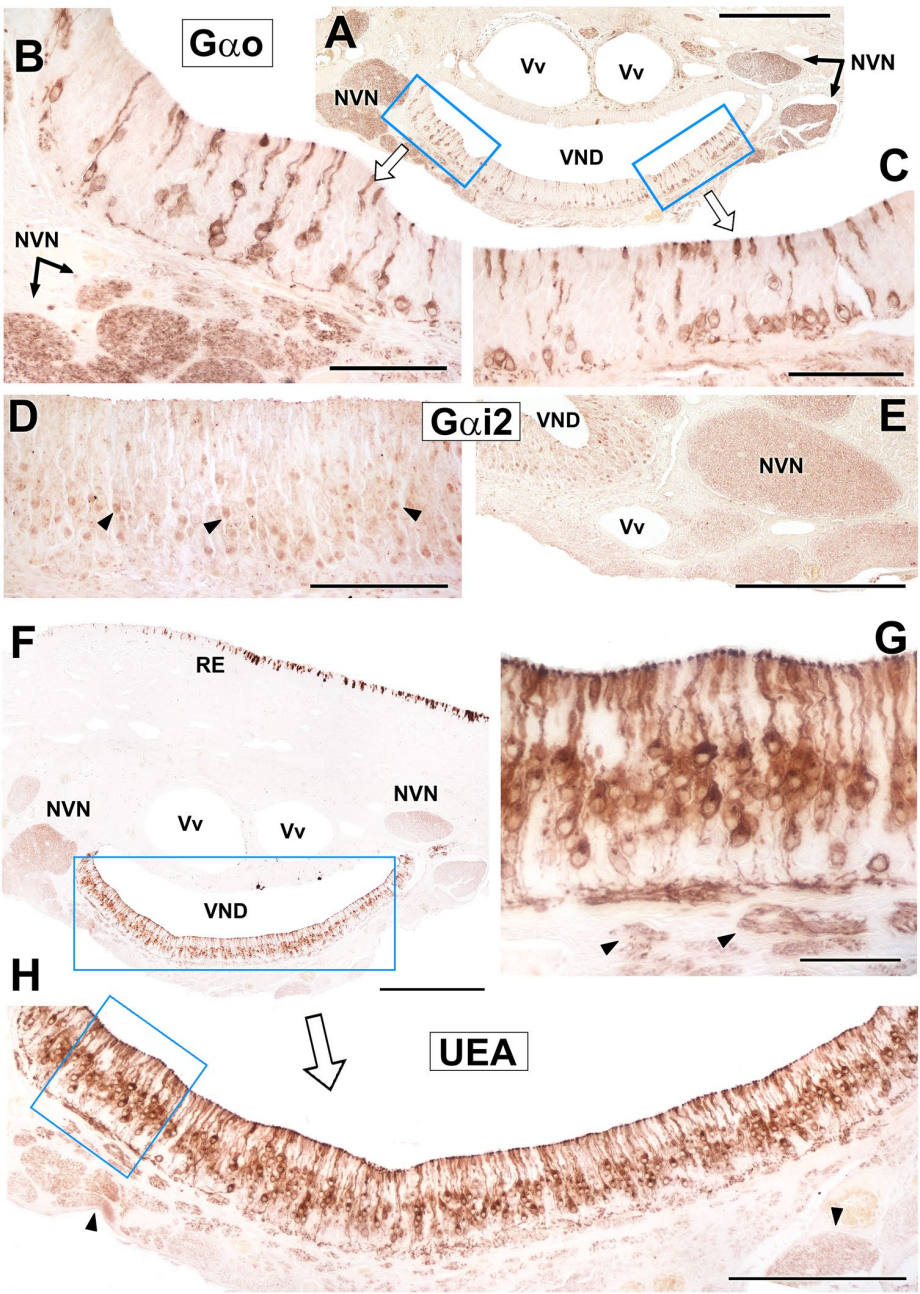
Immunohistochemistry and lectin histochemistry of the Bennett’s wallaby VNO. **(A)** Immunolabelling against Gαo in the vomeronasal sensory epithelium (SE). Strong positivity was observed in a subpopulation of neuroreceptor cells along the entire epithelium. **(B and C)** Insets in A show the labelling comprising the neuroreceptor cell somas, dendrites running to the luminal surface, and axons coursing towards the lamina propria. **(D, E)** Anti-Gαi2 immunolabelling is concentrated in the somas of the neuroreceptor cells (arrowheads). The positivity is confirmed at the level of the vomeronasal nerves (NVN). (**F–H)** Images at different magnifications showing histochemical labelling with *Ulex europaeus* agglutinin (UEA) lectin in the neuroepithelium. The labelling is concentrated in the somas and dendrites, as well as in the lamina propria nerve bundles (arrowheads in G and H). NVN: Vomeronasal nerve; RE: Respiratory epithelium; SE: Sensory epithelium; VND: Vomeronasal duct; Vv: Veins. Scale bars: (A, E, F) 500 µm; (H) 250 μm; (B, C, D) 100 μm; (G) 50 µm

Immunostaining of the VNO sensory epithelium using antibodies against the Gαo and Gαi2 proteins shows characteristic patterns. Gαo neuroreceptor cells are not excessively abundant but are labeled very neatly and with great intensity in the pericarion and along the dendritic and axonal prolongations. The soma are distributed along the entire thickness and perimeter of the neuroepithelium (Fig. 10A–C). Gαi2 cells are more numerous, but the labeling is less striking as it is primarily concentrated in the somas (Fig. 10D). For both markers, strong immunopositivity is observed in the vomeronasal nerves (Fig. 10A, B, E). Histochemical labeling with UEA lectin stains the entire receptor epithelium (Fig. 10F), although without labeling the entire population of neuroreceptor cells. UEA lectin produces an intense and sharp marking that affects both the dendrites in the luminal area of the sensory epithelium and the axons in the lamina propria (Fig. 10G, H).

**Fig. 11:**
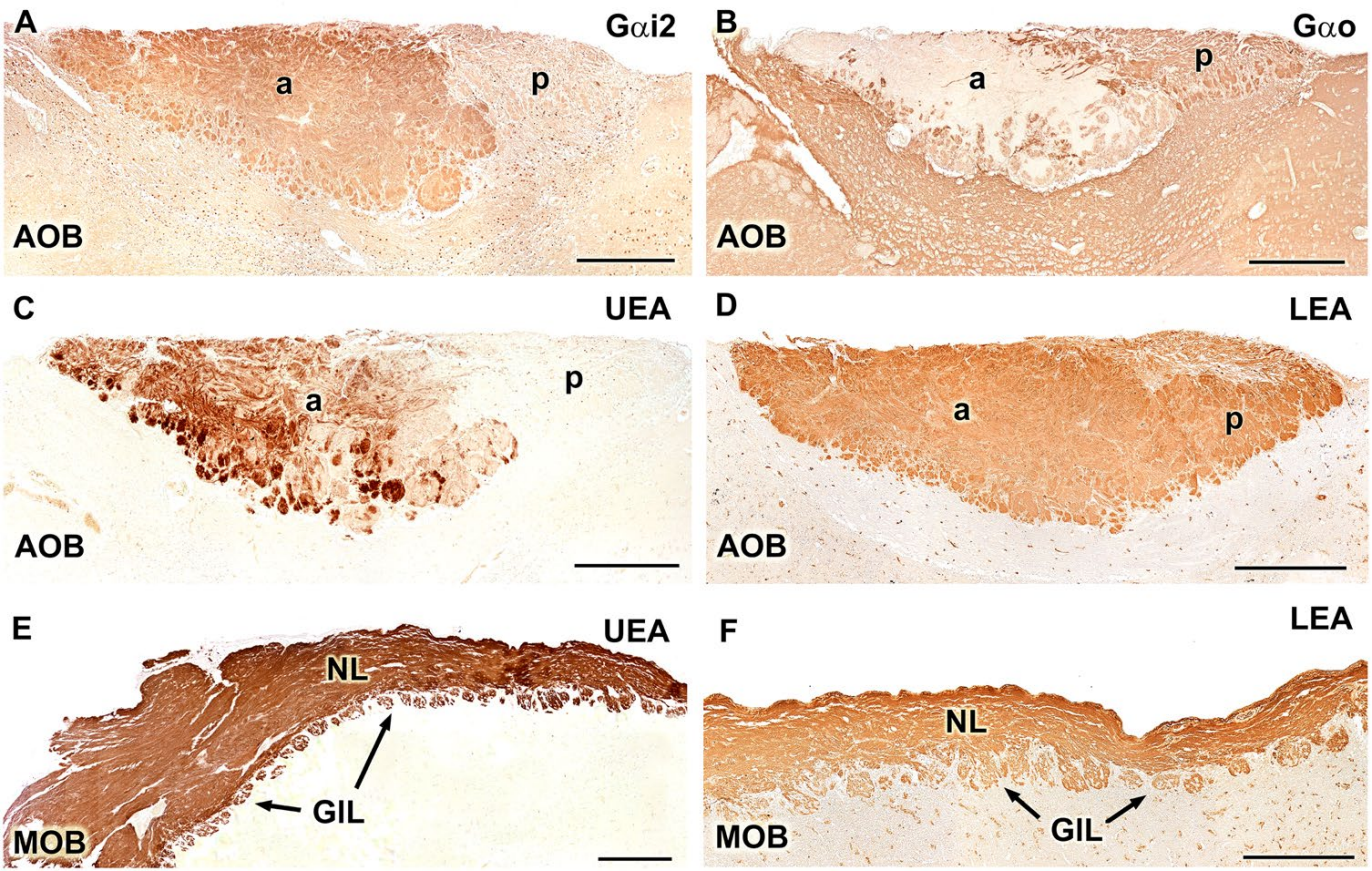
Immunohistochemical and lectin-histochemical labelling of the Bennet’s wallaby AOB. **(A)** Anti-Gαi_2_ clearly delimits an anterior ( +)/posterior ( −) zonation in the superficial layers of the AOB. **(B)** Anti-Gαo delimits an anterior ( −)/posterior ( +) zonation. **(C and E)** *Ulex europaeus* agglutinin (UEA) lectin labelling confirms the zonation found with anti-Gαi_2_. The glomerular and nerve layers of the main olfactory bulb (MOB) are equally positive for this marker (E). **(D and F)** *Lycopersicon esculentum* agglutinin (LEA) stains the superficial layers of the whole accessory olfactory bulb (AOB, D) and MOB (F).Scale bars: (A, B, C, D) 500 µm; (E, F) 250 µm

The immunohistochemical labeling of G proteins in the AOB allowed us to confirm that the anteroposterior zonation suggested by the Nissl staining also reflects the differential expression of G proteins in the superficial layers of the AOB. Specifically, the anterior half of the VNL-GlL layers in the AOB is Gαi2-positive (Fig. 11A), whereas the posterior half is Gαo-positive, in a complementary manner (Fig. 11B). Additionally, Gαo positivity can be detected in the glomerular layer of the anterior zone. This zonation pattern was confirmed by the use of UEA lectin, which revealed a labeling pattern similar to that for anti-Gαi2, which was circumscribed to the anterior zone (Fig. 11C). UEA is not specific for the AOB and also stains the superficial layers of the MOB (Fig. 11E). The study using the LEA lectin showed the wide positive labeling of the superficial layers of both the AOB (Fig. 11D) and MOB (Fig. 11F).

**Fig. 12:**
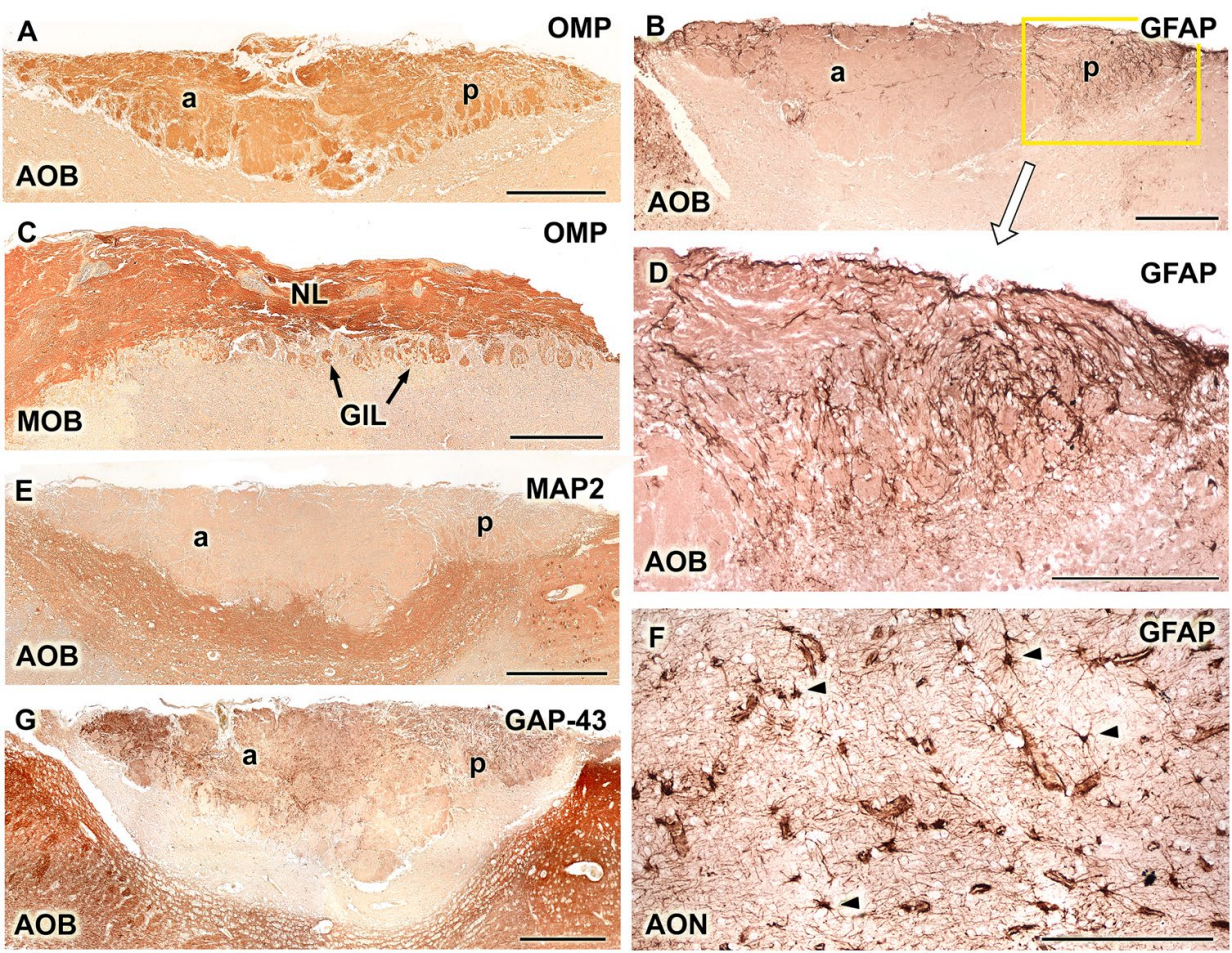
Immunohistochemical labelling of the Bennett’s wallaby AOB. **(A and C)** Anti-olfactory marker protein (OMP) stains the superficial layers of both the accessory olfactory bulb (AOB, A) and the main olfactory bulb (MOB, C). **(B, D, and F)** The anti-glial fibrillary acidic protein (GFAP) labelling is concentrated in the anterior and even more intensely in the posterior (enlarged in D) edges of the AOB. The fibrillar character of the labelling indicates that it corresponds to the ensheathing glia (D). Few astrocytes are observed, although these can be identified in the anterior olfactory nucleus, which serves as positive control**. (E)** Anti-microtubule-associated protein 2 (MAP-2) stains the deeper layers of the AOB: mitral-plexiform and granular. **(G)** Anti-growth-associated protein (GAP)-43 produces positive labelling in the VNL, GlL, and GrL strata. Scale bars: (A, B, E, G) 500 µm; (C, D,F) 250 µm

Anti-OMP stains the superficial layers of both the AOB (Fig. 12A) and MOB (Fig. 12C). Anti-GFAP does not strongly stain the AOB, concentrating most of its labeling in the peripheral regions of the AOB, both anterior and posteriorly (Fig. 12B). In this latter area, anti-GFAP forms a dense network that accompanies the vomeronasal fibers of the nerve layer (Fig. 12D). Remarkably, astrocytes are not observed in the AOB, although they are observed in other areas of the olfactory brain, for instance, the anterior olfactory nucleus (Fig. 12F). Anti-MAP-2 stains the deeper layers of the AOB, the mitral-plexiform and granular layers (Fig. 12E), and anti-GAP-43 labels both superficial layers, nervous and glomerular, in addition to the granular layer (Fig. 12G). However, it does not label the mitral-plexiform zone.

**Fig. 13:**
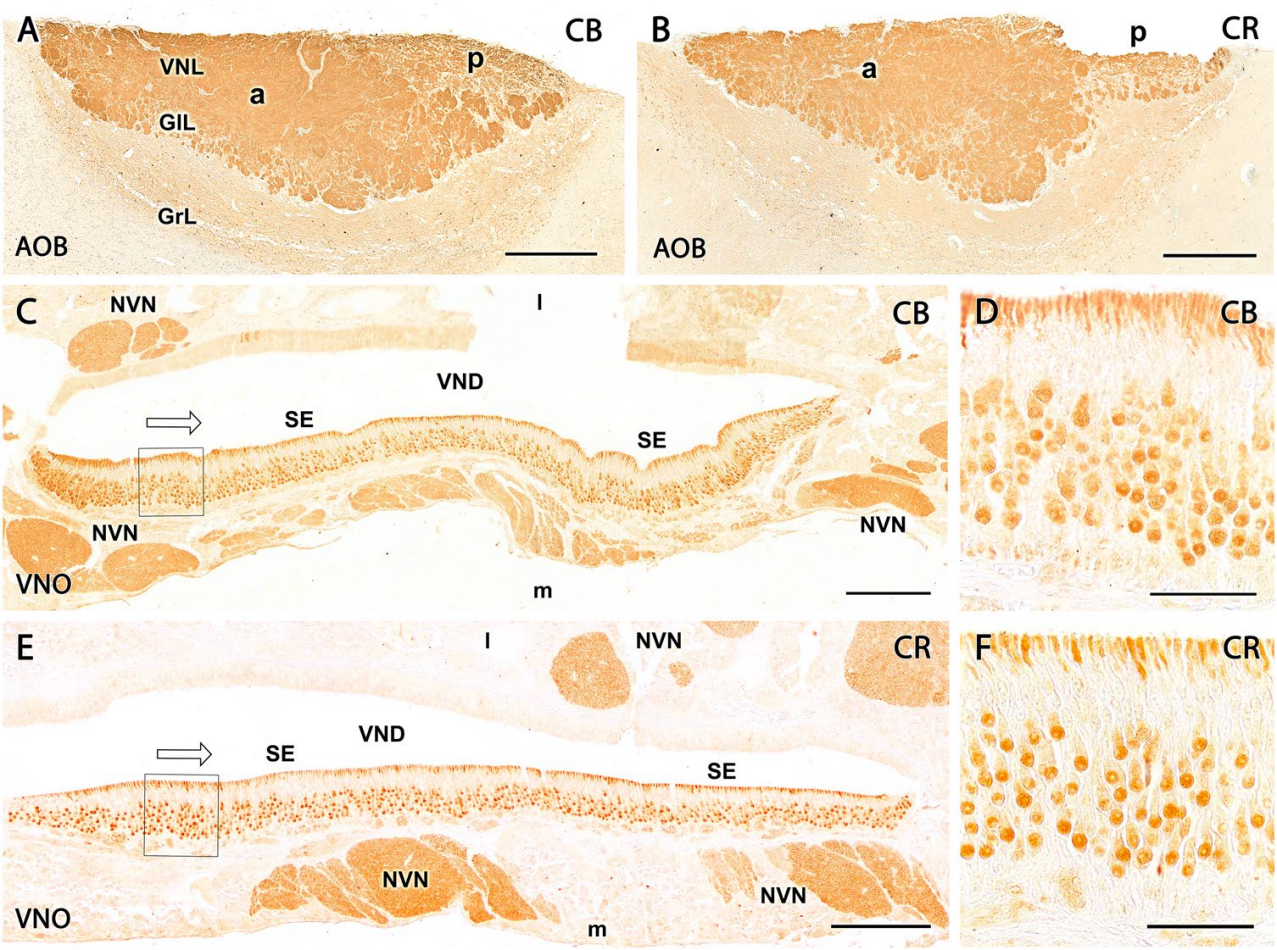
Study of calcium-binding proteins in the Bennett’s wallaby VNS. **(A)** Calbindin and **(B)** calretinin markers show immunopositivity in the neuropil of the nerve and glomerular layers of the AOB. **(C)** In wallaby VNO, anti-calbindin is strongly expressed in NVN and characteristically marks receptor neuroepithelial cells. **(D)** Enlargement of the box shown in C where anti-calbindin produces a label in the nucleus, soma, and dendrites of cells in the central area of the epithelium and less intensely marks some cells in the basal area. **(E)** In the case of calretinin, it produces a marking very similar to that shown by calbindin in the VNO, intensely marking the nerve bundles and **(F)** mainly the cells of the central stratum in the epithelium, although a slight marking is also observed in a certain population of basal cells. Scale bars: (A, B) 500 µm; (C, E) 200 µm; (D, F) 50 µm

The calcium-binding proteins calbindin (CB) and calretinin (CR) are expressed along the whole VNS. In the AOB, both antibodies labeled the NVN and glomerular layer of the AOB in a diffuse and strong manner (Fig. 13A, B). Neither periglomerular nor mitral cells were immunopositive for either of these markers. Individual somas were observed for both markers in both the mitral-plexiform and granular layers of the AOB.

In the VNO neuroepithelium, both markers produced similar labeling patterns, characterized by the intense staining of the axonal bundles of the NVN and most of the neuroreceptor cells. The labeling was concentrated in the dendritic knobs and soma (Fig. 13C–F).

## Discussion

The study of the expression patterns of the α-subunit of the Gi2 and Go proteins has been crucial to understanding the mechanisms of vomeronasal transduction. Gαi2 serves as a marker for V1R cells, whereas Gαo co-expresses with V2R cells. In the AOB, the NVNs express both G proteins according to a topographical anteroposterior zonation in which the anterior region expresses Gαi2 and the posterior region expresses Gαo. This segregated model is typical of species belonging to the orders Rodentia, Lagomorpha, Afrosoricida, and Didelphimorphia. A deterioration in the Gαo pathway is observed in species belonging to orders as diverse as Soricidae, Hyracoidea, Perissodactyla, Artiodactyla, Carnivora, and Primates. In these species, the NVN and its termination in the glomeruli only express Gαi2, presenting a uniform model of vomeronasal transduction. This scenario was complicated by the 2012 publication of a study on the VNS in the tammar wallaby, *Notamacropus eugenii*, which unexpectedly revealed that this animal might constitute an alternative model of vomeronasal organization, with vomeronasal neuroreceptor cells along the VNO and AOB expressing only the Gαo protein (Schneider et al. 2012), indicating the existence of a third accessory olfactory type, intermediate to the segregated and uniform types previously described. However, the authors of the study in tammar wallaby warned that their findings should be taken with caution, as some of their observations prevented the drawing of definite conclusions. Very atypically for a uniform model of G protein subunit expression, the anti-Gαo protein was only expressed in a subpopulation of neuroreceptor cells in the vomeronasal epithelium rather than in the whole population of VR neurons. To explain this low number of Gαo-positive cells, the authors hypothesized a reduced affinity of the antibody used for the tammar Gαo; however, no other examples of any similar selectivity deficits in response to the use of anti-Gαo antibodies have been described in the literature.

More critically, immunolabeling of the tammar wallaby AOB using anti-Gαo has only been demonstrated by this single study (Fig. 6E in Schneider et al. 2012). Their staining protocol resulted in very faint labeling, which was restricted to a very small area of the superficial layers of the AOB receiving projections from the vomeronasal neuroreceptor cells. In contrast, in all other studied species, in both the segregated and uniform models, both Gαo and Gαi2 antibodies are associated with positive immunolabeling that can be observed throughout the entire thickness of the superficial layers, including the NVN and glomerular layers. Regrettably, the authors do not specify the antibody employed that resulted in a Gαi2 immunonegative pattern in both the VNO and AOB. Neither the commercial source nor the antibody lot number was reported, precluding any comparisons with other studies of Gαi2 expression in the VNS using the same antibody.

To date, no further studies have been reported that either reinforces or rejects the potential existence of this third model of organization for the vomeronasal transduction pathways. Therefore, we have performed a morphofunctional study of the VNS of the Bennett’s wallaby (*Notamacropus rufogriseus*) to characterize the expression patterns of G proteins in this species to shed light on the proposed presence of a third model of vomeronasal transduction in macropodids.

Our immunohistochemical study of G proteins in the Bennet’s wallaby revealed a canonical immunohistochemical labeling pattern for both Gαi2 and Gαo in the VNO and AOB of all samples studied. Moreover, the pattern of labeling observed in the Bennett’s wallaby vomeronasal neuroepithelium using anti-Gαo staining (Fig. 10A–C) was identical to that described for the tammar wallaby (Fig. 3A and B in Schneider et al. 2012), in which only a small fraction of neuroreceptor cells were labeled. The nuclei of these cells were distributed along the entire thickness of the neuroepithelium, with very strong staining in the somas and the knob-like structures protruding into the VNO lumen from the receptor cell endings. However, whereas Schneider et al. did not obtain positive immunolabeling with their anti-Gαi2 antibody in the VNO, we observed an abundant number of immunopositive cells in the neuroepithelium (Fig. 10D) using our anti-Gαi2 antibody. The strong immunopositivity identified in the vomeronasal axons in both the lamina propria and the nasal mucosa confirmed the neuroreceptor nature of these cells (Fig. 10E).

By extending immunohistochemical characterization of G protein expression to the AOB, we were able to verify the existence of the anterior–posterior zonation pattern that is typical of mammalian species belonging to the segregated model. The vomeronasal axons reaching the anterior zone of the AOB only expressed Gαi2, whereas those reaching the posterior zone only expressed the Gαo subunit, and the immunolabeling of these two G proteins revealed a complementary pattern. Gαo positivity was also observed in the glomerular layer of the Gαi2-positive anterior zone, a layer that contains many dendritic projections from mitral and other neurons located in deeper layers of the anterior region, similar to the observations described by Takigami et al. (2004), Villamayor et al. (2018), and Torres et al. (2020). The observed Gαo-positivity is, therefore, likely due to the presence of projections from Gαo-positive cells located in the anterior part of the neuropil onto glomeruli found in this area; however, because these projections do not originate from vomeronasal axons, this anterior glomerular Gαo-positive labeling cannot be considered a justification for the uniform model. The employment of the UEA lectin provided additional evidence for the segregation of vomeronasal information in the wallaby, revealing the selective labeling of the anterior zone of the AOB. The affinity of the UEA lectin for the anterior AOB has been reported for all the species belonging to the segregated model in which this histochemical marker has been investigated, including hamster (Taniguchi et al. 1993), mouse (Salazar et al. 2001; Salazar and Sanchez-Quinteiro 2003; Kondoh et al. 2017a, b), rat (Salazar and Sanchez Quinteiro 1998), and capybara (Torres et al. 2020). However, studies using UEA lectin staining in species belonging to the uniform model, including pig (Salazar et al. 2000), cat (Salazar and Sanchez-Quinteiro 2011), and dog (Salazar et al. 2013), and studies that have utilized a very broad panel of lectins, as in the goat (Mogi et al. 2007), have not described any evidence of zonation in the AOB.

The expression patterns observed for both Gαi2 and Gαo proteins in the VNO neuroepithelium, the NVN, and the AOB, including the establishment of a clear zonation pattern that was confirmed by UEA lectin staining, contradict the existence of a hypothetical third model of vomeronasal information processing, as described by Schneider et al. (2012). Although Schneider et al. performed their study in the tammar wallaby, *Notamacropus eugenii*, whereas our study was performed in Bennett’s wallaby, *Notamacropus rufogriseus*, this difference is unlikely to explain the observed differences in immunohistochemical labeling, as differences in both the structure and G protein expression patterns of the AOB have never been reported between species belonging to the same genus or family (Meisami and Bhatnagar 1998; Halpern and Martinez Marcos 2003).

Moreover, the main morphological and histological features of the VNS described for *Notamacropus eugenii* are indistinguishable from those found by us in *Notamacropus rufogriseus*. Thus, comparing the observations of Schneider et al. for the VNO (2008) and AOB (2012) of *M. eugenii* with our observations in *M. rufogriseus* reveals that both species share important aspects, such as the opening of the VNO to the nasopalatine duct, the semilunar shape of the VND, with the presence of a remarkable mushroom body, the layering and cellularity of the neuroreceptor epithelium, and the presence of many PAS-positive vomeronasal glands in the lateral parenchyma. Both species share a comparable arrangement of large blood vessels surrounding the VND along its medial and lateral planes, and a similar presence of profuse dorsal, medial, ventral, and ventrolateral unmyelinated innervation is described for both species (Fig. 3B in Schneider et al. 2008), transmitting the information collected by the vomeronasal sensory neuroepithelium to the AOB. This arrangement of vomeronasal axons in the parenchyma is very atypical and has not previously been described in the VNO of any other mammal species apart from these two Macropodidae.

In both species, all the nerve bundles leave the VNO through a dorsolateral opening in the vomeronasal capsule, which is cartilaginous in both species. Another feature common to the VNO of both macropodids, which was observed by Schneider et al. (2008) and Sanchez-Villagra (2001) in the tammar wallaby and which we have verified in our histological series characterizing Bennett’s wallaby, is the failure of the caudal VNC enclose the entire VNO, such that the posterior portion of the VNO is free of surrounding cartilage. A similar finding has been reported for other marsupials, such as *Notoryctes* (Sweet 1904) and *Caenolestes* (Broom 1926).

The information available for the AOB of the tammar wallaby is limited to the study of its lamination using hematoxylin–eosin and Nissl stains and the expression pattern of Gα subunits (Schneider et al. 2012). Our work extends these histological observations through the additional use of Tolivia staining, which allows for the characterization of the AOB lamination with greater definition, including its relationship with the lateral olfactory tract and the organization of mitral cells, all of which appeared comparable in the Bennet’s wallaby to species with highly developed AOB structures, such as rodents (Meisami and Bhatnagar 1998) and Lagomorpha (Villamayor et al. 2020). Similarly, our immunohistochemical study allowed for the characterization of UEA and LEA lectin affinity and the immunohistochemical characterization using various marker proteins, including OMP, MAP2, GAP43, and GFAP, which were performed for the first in this marsupial family.

The UEA labeling, we previously discussed as a differential marker for the anterior zone of the AOB, with a distribution pattern analogous to that of Gαi2, is not specific to the VNS of the Bennett’s wallaby, as staining of the superficial layers of the MOB was also observed, similar to the distribution observed in rodents, such as the capybara (Torres et al. 2020), or in pigs (Salazar et al. 2000). LEA lectin stains all mammals, which has been investigated in the vomeronasal and olfactory neuroepithelia (Park et al. 2012; Lee et al. 2016) and the nerve and glomerular layers of both the AOB and MOB compartments in the mouse (Salazar et al. 2001), sheep, pig (Salazar et al. 2000), and rabbit (Villamayor et al. 2020). In the wallaby, unlike UEA, LEA produces a labeling pattern without zonation, similar to the pattern observed for OMP, a marker of mature olfactory and vomeronasal cells (Bock et al. 2009), and identical to the LEA pattern observed in the rabbit AOB, a species in which the zonation determined by G proteins cannot be discriminated using LEA and OMP (Villamayor et al. 2020).

Anti-GAP-43 is one of the best-characterized markers of growing and regenerating neuronal processes (Ramakers et al. 1992) and was able to identify growing axons in the Bennet’s wallaby AOB, with no difference observed between the anterior and posterior zones. However, the antibody against GFAP showed stronger immunolabeling of glial components in the posterior portion of the AOB relative to the anterior segment, a pattern that has not been previously reported for other studies of this marker in the mammalian AOB (Salazar et al. 1994, 2006) and should be examined in more detail in future studies.

CB and CR are expressed in the entire VNS, along the VNO neuroepithelium, the NVN, and AOB. For both markers, the immunostaining of the VNO comprises the soma and dendrites in a pattern similar to that described in the mouse (Kishimoto et al. 1993). Additionally, in rats, CR labels most of the neuroreceptors cells, whereas CB only labels a neuronal subpopulation (Jia and Halpern 2003). The distribution of CB and CR immunoreactivity in the Bennett’s wallaby’s AOB concentrates on the vomeronasal fibers and glomeruli in a pattern analogous to that found in other species, such as the rabbit (Villamayor et al. 2020) or the capybara (Torres et al. 2020). In the case of other marsupials, such as the opossum, striking differences in the labeling patterns for CB and CR in the AOB were observed compared with the pattern found in the wallaby. In the opossum AOB, CB-labeled neurons were found in all layers except the nerve layer and the periglomerular cells, whereas CR showed a pattern analogous to that found in the wallaby, although in opossum, CR also labels mitral cells and discriminates an anteroposterior zonation, with more intense staining in the posterior AOB than in the anterior AOB zone (Jia and Halpern 2004).

Overall, the VNS of the Bennett’s wallaby shows a degree of differentiation and histochemical and neurochemical diversity comparable to species with greater VNS development. The existence of the third intermediate type in vomeronasal information processing reported in *Notamacropus eugenii* is not supported by our lectin-histochemical and immunohistochemical findings in *Notamacropus rufogriseus*. We confirm the presence of effective expression of the two primary VR families in the VNS of the Bennett’s wallaby, and our contribution expands the morphological, histochemical, and immunohistochemical information available on the VNS of Macropodidae and marsupials in general.

## Supplementary Information

Below is the link to the electronic supplementary material.

Supplementary file1 Suppl. Fig. 1. Glandular tissue of the VNO. (A) Gallego's trichrome staining in the dorsolateral part of the vomeronasal organ. The glandular tissue (arrowheads) surrounds the venous sinuses (Vv). Glands open in the dorsal commissure of the vomeronasal duct (arrow). (B and C) Histochemical study of the dorsolateral vomeronasal glands. Those closer to the vomeronasal duct are strongly periodic acid–Schiff (PAS)-positive (black arrowheads in C) and Alcian blue–negative (black arrowheads in B). However, the more dorsal glands are Alcian blue–positive (white arrowhead in B) and PA-negative (white arrowhead in C). NVN: Vomeronasal nerve; SE: Sensory epithelium. Scale bar: (A,B,C) 100 µm (TIF 18921 KB)

Supplementary file2 Suppl. Fig. 2. Transverse section of the VNO at its more caudal level stained with PAS. The cul-de-sac ending of the vomeronasal duct (VND) has an irregular feature. The whole area is rich in periodic acid–Schiff (PAS)-positive tubule-acinar tissues (VNG) and non-myelinated branches of the vomeronasal nerves (asterisks). The venous sinuses (Vv) are smaller than in the central part of the vomeronasal organ (VNO), and there are small arteries (for instance, the artery in the yellow inset, which is shown at higher magnification in Fig. 8D of the manuscript. Scale bar: 500 µm (TIF 16740 KB)

## Data Availability

Not applicable.
