# Supplementary figures and images for: Does a third intermediate model for the vomeronasal processing of information exist? Insights from the macropodid neuroanatomy
Source: Brain Struct Funct. 2021 Nov 20;227(3):881–99. doi: 10.1007/s00429-021-02425-2 (PMC8930919; doi:10.1007/s00429-021-02425-2)

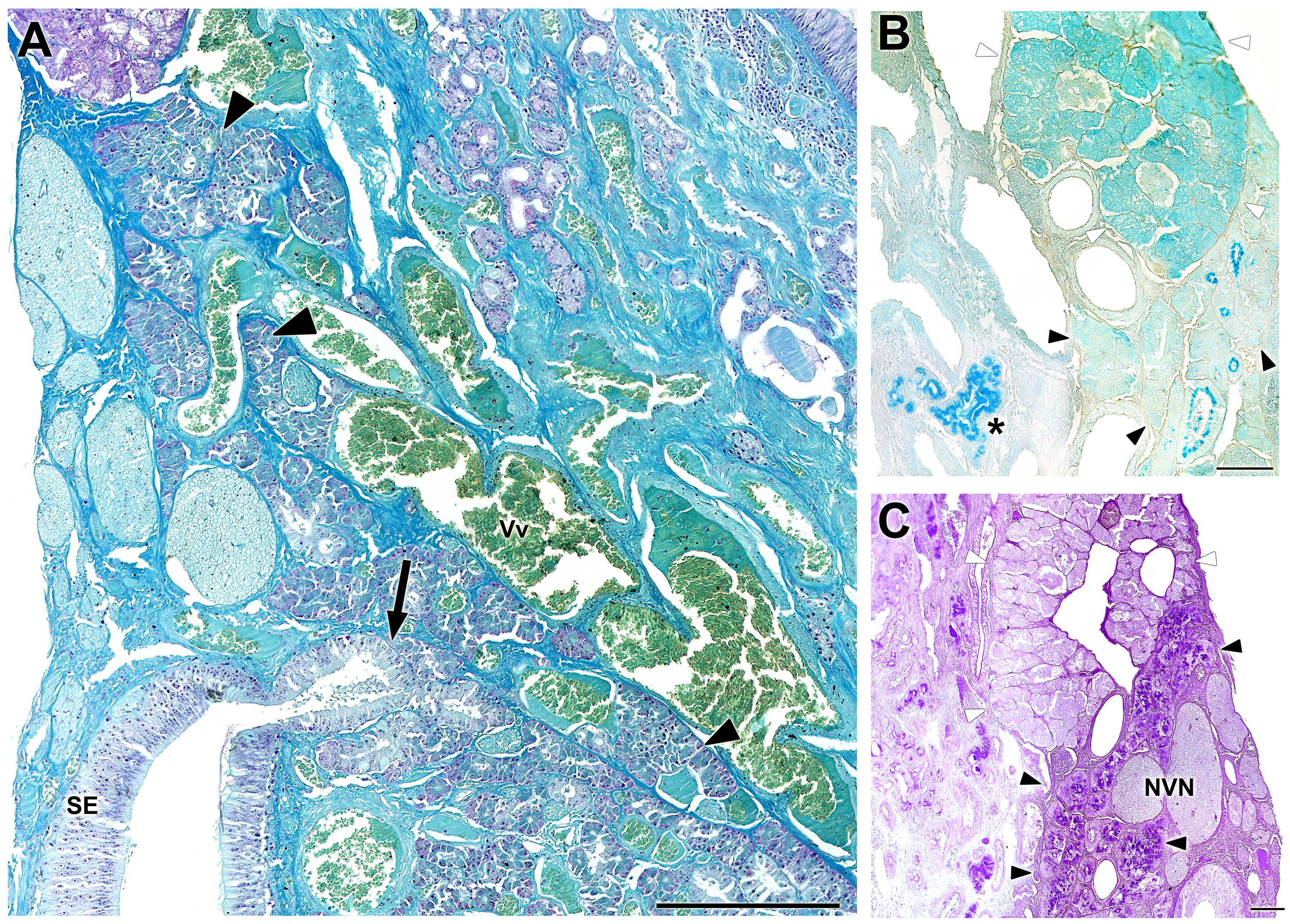

Supplement: Supplementary file 1 — Supplementary file1 Suppl. Fig. 1. Glandular tissue of the VNO. (A) Gallego's trichrome staining in the dorsolateral part of the vomeronasal organ. The glandular tissue (arrowheads) surrounds the venous sinuses (Vv). Glands open in the dorsal commissure of the vomeronasal duct (arrow). (B and C) Histochemical study of the dorsolateral vomeronasal glands. Those closer to the vomeronasal duct are strongly periodic acid–Schiff (PAS)-positive (black arrowheads in C) and Alcian blue–negative (black arrowheads in B). However, the more dorsal glands are Alcian blue–positive (white arrowhead in B) and PA-negative (white arrowhead in C). NVN: Vomeronasal nerve; SE: Sensory epithelium. Scale bar: (A,B,C) 100 µm (TIF 18921 KB) [file 429_2021_2425_MOESM1_ESM.tif]

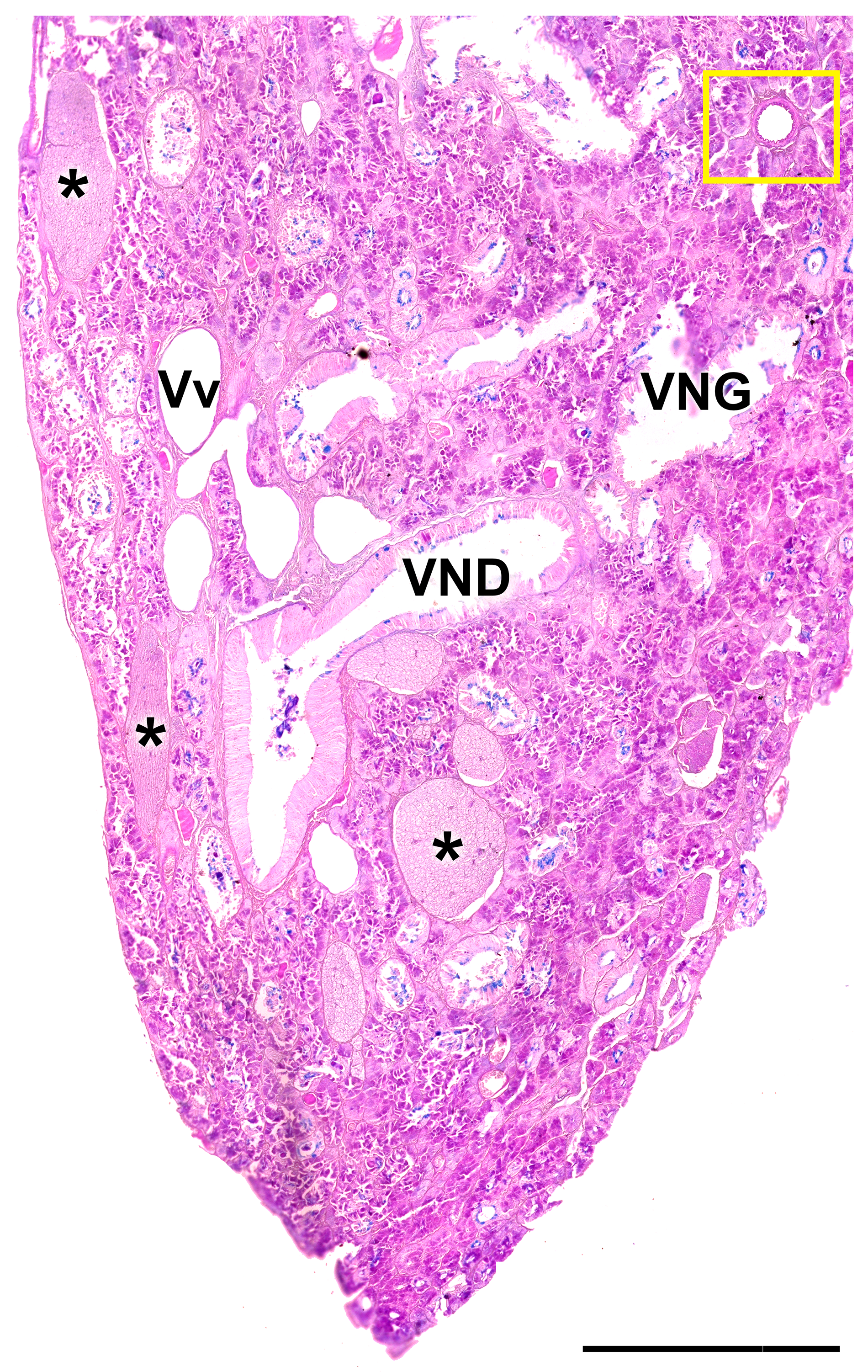

Supplement: Supplementary file 2 — Supplementary file2 Suppl. Fig. 2. Transverse section of the VNO at its more caudal level stained with PAS. The cul-de-sac ending of the vomeronasal duct (VND) has an irregular feature. The whole area is rich in periodic acid–Schiff (PAS)-positive tubule-acinar tissues (VNG) and non-myelinated branches of the vomeronasal nerves (asterisks). The venous sinuses (Vv) are smaller than in the central part of the vomeronasal organ (VNO), and there are small arteries (for instance, the artery in the yellow inset, which is shown at higher magnification in Fig. 8D of the manuscript. Scale bar: 500 µm (TIF 16740 KB) [file 429_2021_2425_MOESM2_ESM.tif]
